# Rare genetic diseases associated with G-quadruplex-induced replication stress

**DOI:** 10.1038/s42003-026-09966-4

**Published:** 2026-04-13

**Authors:** Lauren M. Herr, Swagata Mukhopadhyay, Olivia M. Anderson, Tucker H. Couch, Cate M. Jones, Robert M. Brosh, Martina Rossi

**Affiliations:** Helicases and Genomic Integrity Section, Translational Gerontology Branch, National Institute on Aging, NIH, Biomedical Research Center, Baltimore, MD USA

**Keywords:** DNA, Genomic instability, Cancer, DNA metabolism

## Abstract

DNA replication stress is incurred by endogenous or environmental challenges to replication fork progression that impede faithful genome duplication. Genomic G-quadruplexes (G4s) are DNA secondary structures that present a substantial barrier for passage of the replisome, and DNA synthesis past these structures requires dynamic remodeling by specialized helicases, translocases, and other G4-binding proteins to facilitate G4 resolution or bypass. Mutations in the genes encoding these auxiliary replication proteins are linked to hereditary disorders presenting with a range of clinical features, including immunodeficiency, growth restriction, congenital abnormalities, and cancer predisposition, demonstrating that these G4-metabolizing proteins also play broader roles in genome biology such as the replication stress response or DNA repair. Here, we review rare diseases linked to mutations in G4-resolving and binding proteins, with an emphasis on molecular defects in G4 metabolism that incur replication stress and genomic instability. We discuss differences in G4 substrate specificity and mechanism of G4-interactive helicases, as revealed by high-resolution structural data. Furthermore, we address outstanding questions that provide insight into the etiology of rare diseases marked by dysregulated G4 homeostasis and may inform diagnosis and potential therapeutic strategies.

## Abstract (web summary)

The authors review the consequences of mutations in genes linked to rare hereditary disorders associated with G-quadruplex (G4)-induced replisome stalling, as well as the molecular functions and cellular pathways of these G4-interacting proteins.

## Introduction

Long evidenced to form in guanine (G)-rich nucleic acid sequences, G-quadruplexes (G4s) are non-canonical nucleic acid structures composed of stacked planar G-quartets formed by Hoogsteen base-pairing of guanine residues, which can assemble into a variety of topologically diverse intra- and intermolecular conformations^1,2^. Over 370,000 sequences in the human genome have been computationally determined to have G4-forming potential by the presence of the consensus formula G_3+_N_1-7_G_3+_N_1-7_G_3+_N_1-7_G_3+_^3^. More recently, over 700,000 G4s have been detected in the human genome using high-resolution sequencing, including previously overlooked, non-classical structures containing long loops and bulges^4^. Sequencing approaches have revealed enrichment of G4 motifs in gene regulatory elements such as promoters, 5′-untranslated regions, and splicing sites^4^, and G4s have been directly detected at telomeres using the structure-specific antibody BG4^5,6^, developed by the Balasubramanian lab. Binding of G4 structures by transcription factors, methyltransferases, and subunits of the shelterin complex implicate G4s in regulation of transcription, epigenetic modulation, and telomere homeostasis^2^.

In addition to intrinsic sequence composition and presence of stabilizing monovalent cations, genomic G4 formation and stability are influenced by the structural context of the surrounding chromatin environment^2^. Due to competition with standard Watson-Crick base-pairing, G4 formation is favored in the context of single-stranded DNA (ssDNA)^7–9^ and is thus promoted by dynamic genomic transactions that necessitate strand separation of duplex DNA such as replication, recombination, and transcription. G4 formation and stabilization are supported by non-canonical structures formed during these processes that occupy the complementary strand, such as transcriptional R-loops, three-stranded nucleic acid structures consisting of a DNA-RNA hybrid with a displaced DNA complementary strand^10^. Consistently, G4s have been observed to be genomically enriched in nucleosome-depleted regions harboring actively transcribed genes in human cells by BG4 chromatin immunoprecipitation (ChIP)-sequencing^11^.

The genomic DNA G4 landscape is actively modulated by G4-stabilizing proteins and DNA helicases that may regulate the physiological processes in which these structures are implicated^2,12^. Dynamic formation and resolution of G4 structures is critical for genomic transactions such as DNA replication, a vital process that ensures the faithful passage of genetic information during every cell division. Cellular DNA replication is catalyzed by a eukaryotic multi-protein complex known as the replisome which consists of both stable and transiently interacting factors. Replication progression may be impeded by a variety of endogenous or exogenous stressors, such as physical barriers to fork advancement that include DNA lesions, secondary DNA structures, and protein complexes such as transcription machinery, as well as genetic deficiencies in replication proteins that disrupt replisome assembly or fork stability^13^. Unfolding G4 structures requires mechanical forces greater than those most replicative DNA polymerases can exert^14–17^. Although some translesion DNA polymerases can bypass G4 structures to aid in efficient genome replication, a number of replicative DNA polymerases require coordinate action with G4-resolving helicases to capably catalyze DNA synthesis^18^. Thus, helicases serve as important auxiliary replication factors that facilitate fork progression through G4-forming sequences such as telomeres that pose a challenge to replication machinery^19,20^.

Disruption of efficient and faithful completion of DNA replication, known as replication stress, can result in DNA damage accrual, chromosomal instability, cell cycle interference, mutagenesis, and cellular senescence, which contribute to aging, cancer, and disease^21^. Irreversible mutations resulting from repair defects can have deleterious consequences on gene expression efficiency or fidelity. The detrimental impact of impaired replication progression/completion for organismal health is evidenced by an extensive list of hereditary disorders linked to mutations in replisome subunit-encoding genes marked by cellular phenotypes of replication stress^22^. Notably, mutations in G4-metabolizing proteins are also linked to rare diseases that in many cases display clinical features and cellular phenotypes reflective of G4-induced replication stress (Table 1). A rare disease is defined by its prevalence in the general population. The frequency of a rare disease varies based on its classification as a rare, ultra-rare, or hyper-rare with estimates varying from ≤1/10^3^ to ≤1/10^6^ people^23^. Notably, single-gene mutations characterize the majority of rare diseases, with approximately 80% of all rare diseases having a genetic origin attributable to monogenic disorders^24^.

**Table 1:** Rare hereditary diseases linked to genetic defects characterized by G4-induced replication stress^a^

Rare hereditary disease	Gene	Clinical features	Prominent cellular deficiencies, highlighting G4 metabolic defects
Bloom syndrome (BS)	BLM	Growth deficiency; sun-sensitivity; high cancer risk; abnormal immune response	BLM deficiency causes reduced DNA synthesis rate, accumulation of abnormal DNA replication intermediates, hypersensitivity to agents that induce replication stress, and inaccurately processed late-replicating DNA intermediates; BLM-deficient cells display elevated sister chromatid exchange enriched at G4-forming motifs in transcribed genes, reduced telomeric guanine-rich strand DNA synthesis, altered expression of genes with G4-forming potential, and stress granule-associated RNA-G4 accumulation
DNA2-related mitochondrial DNA deletion syndrome	DNA2^b^	Progressive external ophthalmoplegia; ptosis; muscle weakness	DNA2 deficiency causes G4 accumulation, replication stalling, fragile telomeres, mitochondrial DNA instability and oxidative phosphorylation defects, aberrant splicing, and chromosomal instability
Seckel syndrome type 8	DNA2^b,c^	Microcephaly; dwarfism	
Dyskeratosis congenita (DC)	RTEL1^b,c^	Bone marrow failure; oral leukoplakia; reticular skin pigmentation; abnormal nail formation	RTEL1 deficiency leads to short telomeres, R-loop accumulation, replication defects, and G4 enrichment
Hoyeraal–Hreidarsson syndrome (HHS)	RTEL1^b,c^		
Fanconi anemia (FA)	FANCJ (BRIP1/BACH1)^c^	Bone marrow failure; abnormal digits and stature; predisposition to cancer, especially head and neck; malformation of organs	FANCJ deficiency causes hypersensitivity to DNA cross-linking agents and G4 ligands as assayed by cell viability, apoptosis, DNA damage induction, and replication defects; FANCJ-deficient cells display G-tract instability and G4 accumulation
Fanconi anemia (FA)	FANCD1 (BRCA2)^c^		FANCD1/BRCA2 deficiency alters G4-related telomeric homeostasis
Myelodysplastic syndrome (MDS)	HLTF	Blood cytopenia; bone marrow dysplasia; acute myeloid leukemia	HLTF deficiency causes accumulation of DNA damage, double-strand breaks, and reduced PCNA polyubiquitylation via decreased binding with MMS2 and UBC13
RECON syndrome	RECQL1	Progeroid-like facial abnormality; skin photosensitivity and xeroderma; growth retardation; dry eyes; delayed eruption of permanent teeth	RECQL1 deficiency causes impaired response to topoisomerase inhibition, replication stress
Rothmund–Thomson syndrome (RTS)	RECQL4^b^	Poikiloderma; juvenile cataracts; short stature; thin hair and eyebrows/eyelashes; nail dysplasia; skeletal abnormalities; osteogenic sarcomas	RECQL4 deficiency confers elevated DNA damage and genomic instability, hypersensitivity to oxidative stress, telomere abnormalities, and G4 accumulation at telomeres
Baller–Gerold syndrome (BGS)	RECQL4^b^	Radial aplasia/hypoplasia; craniosyntosis	
RAPADILINO syndrome	RECQL4^b^	Radial aplasia/hypoplasia; patellae hypoplasia; cleft or highly arched palate; diarrhea; dislocated joints; small size; limb malformation	
Warsaw Breakage. syndrome (WABS)	DDX11	Microcephaly; pre- and post-natal growth retardation; abnormal skin pigmentation; scleroderma; short stature; pinched facial features	DDX11 deficiency causes reduced replication fork progression, impaired cohesin binding to chromatin during S-phase, and reduced fork speed after G4 ligand exposure
Werner syndrome (WS)	WRN	Bilateral ocular cataracts; greying and loss of hair; scleroderma; cardiovascular disease; sarcomas; Type II diabetes; osteoporosis	WRN-deficiency causes prolonged S-phase, reduced DNA synthesis initiation, fork progression, and fork recovery after DNA damage, defective telomere lagging strand DNA synthesis, hypersensitivity to DNA damaging agents and G4 ligands, and poor recovery of replication forks stalled by G4 ligand stabilization; WRN deficiency alters expression of genes with G4-forming potential

^a^See text for details.

^b^Mutations in the same gene are linked to distinct clinical diseases.

^c^Mutations in additional genes other than those listed are linked to the designated genetic disorder, but these additional genes are not directly implicated in G4 metabolism.

Here, we review the consequences of mutations in genes linked to rare hereditary disorders characterized by aberrant genome homeostasis, with a focus on cellular phenotypes reflective of replication stress and genomic instability induced by G4 DNA. We discuss cellular and clinical phenotypes of rare diseases linked to replisome components and present recent evidence for G4-induced replisome stalling in biological systems. We consider the molecular functions and cellular pathways of these G4-interacting proteins in their mechanisms to overcome impeded DNA replication, highlighting the importance of proper G4 metabolism for maintenance of genomic integrity vital to organismal health.

## Replisome functions and genetic defects

Eukaryotic nuclear DNA synthesis is catalyzed by a multi-protein complex known as the replisome, the core of which is composed of Cell Division Cycle 45 (CDC45), Mini-Chromosome Maintenance (MCM) 2-7 subunits, and Go-Ichi-Ni-San (GINS) CDC45 - MCM2-7 - GINS (CMG). The hetero-hexameric ATPase ring composed of seven subunits (MCM2-7) bound to noncatalytic subunits CDC45 and GINS tetramer is physically complexed with the leading strand polymerase epsilon (ε)^25^. A battery of proteins assembles into this replication-competent complex, including the lagging strand polymerases delta (δ) and alpha (α), which work in concert with Flap Endonuclease-1 (FEN1), DNA Replication Helicase-Nuclease 2 (DNA2), and DNA ligase to process Okazaki fragments; fork protection complex factors TIMELESS, TIPIN, CLASPIN, and AND-1; and the polymerase processivity factor Proliferating Cell Nuclear Antigen (PCNA)^26,27^. Additional proteins coordinate replisome assembly and activation, forming the pre-replication and pre-initiation complexes necessary for origin licensing and firing, respectively^28^.

Bi-allelic mutations in the genes encoding the CMG subunits CDC45 and MCM5 have been linked to Meier–Gorlin syndrome, a microcephalic primordial dwarfism characterized by clinical features of short stature, microtia, and aplastic/hypoplastic patellae^29,30^. Growth restriction has also been linked to mutations in *GINS1* and *MCM4*, which present an additional shared clinical feature of natural killer cell deficiency^31,32^. Mutations in *GINS1* are associated with CMG loss of function or instability, and GINS1-deficient patient cells show fewer bidirectional replication forks by single-molecule (SM) DNA fiber analysis, consistent with deficient origin firing, as well as an increase in stalled replication forks^31^. MCM4-deficient cells also show signs of replication stress in the form of DNA damage and chromosomal aberrations following treatment with the DNA polymerase inhibitor aphidicolin^32^. Further, treatment of MCM5-deficient cells with hydroxyurea (HU), which depletes the deoxyribonucleoside triphosphate pool, impairs cellular entrance into S-phase^30^.

In addition to direct mutations in subunits of the CMG helicase, microcephalic dwarfism has been linked to bi-allelic mutations in the replication fork protection factor *DONSON*, which is implicated in CMG helicase assembly and stabilization^33–35^. Clinical features associated with bi-allelic as well as de novo mutations in *DONSON* have been expanded to include skeletal abnormalities^36^. Loss of DONSON leads to severe replication stress, including spontaneous replication fork stalling and asymmetry, as well as elevated S-phase double-strand break (DSB) formation^35^. The array of hereditary disorders and clinical features linked to mutations in both replisome components and proteins that regulate their assembly illuminates the importance of auxiliary replication factors in facilitating effective replication progression and highlights the significant clinical impact of replication stress.

## G4-induced replisome stalling and bypass

**Fig. 1:**
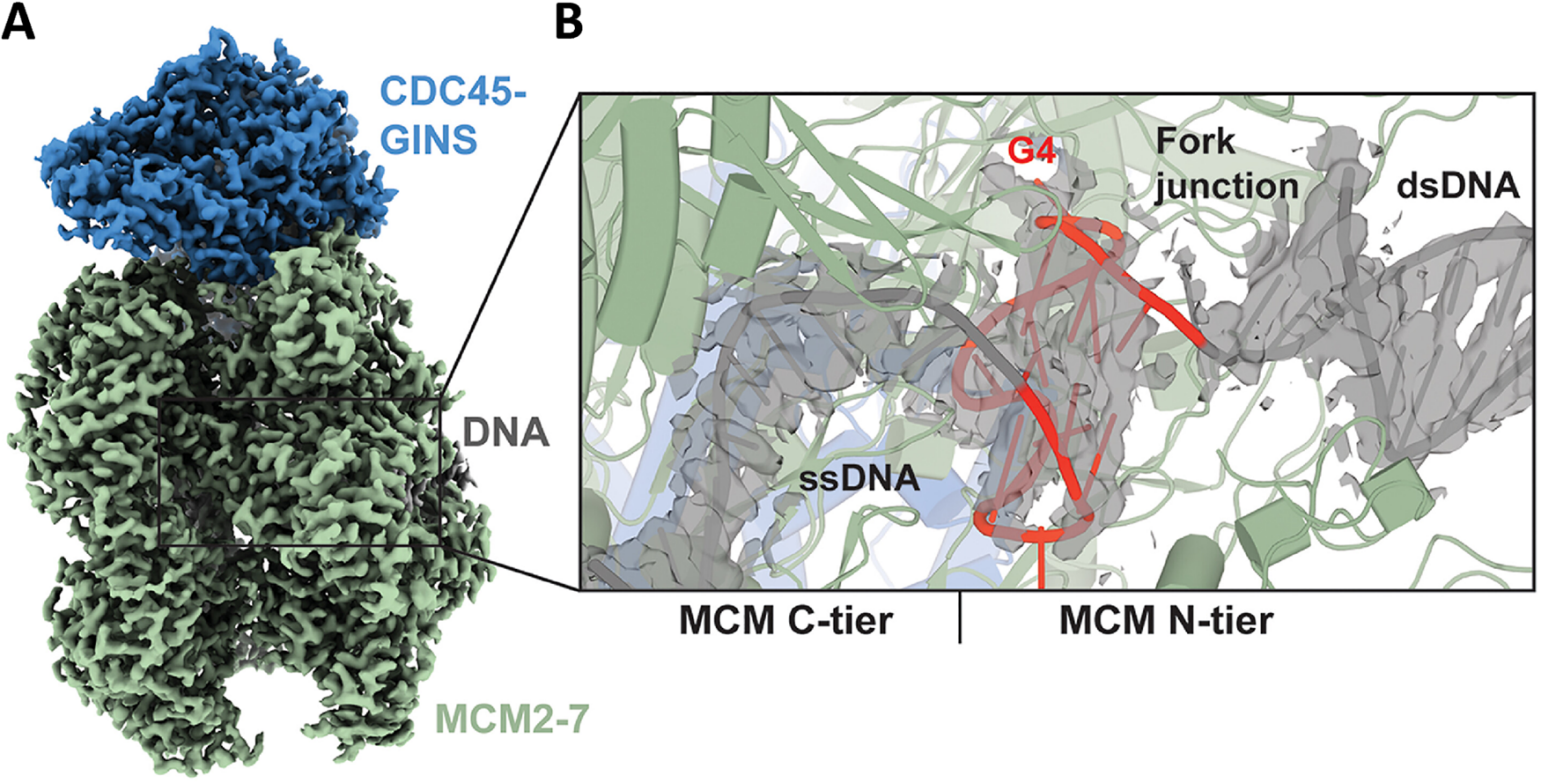
The human replisome is stalled by G4. **A** Cryo-EM density map of human CMG-Fork Protection Complex stalled at G4 colored by subcomplex; **B** Structure of the G4 bound in the central chamber of the MCM ring. From Batra et al., *Science* (2025) 387 (6738) 387. Ref. ^37^. Reprinted with permission from AAAS.

While G4 structures have long been suggested to impede DNA replication, the first report of G4-induced replisome stalling in vertebrates was demonstrated using Xenopus egg extracts, in which the CMG helicase was shown to pause at G4 sites, leading the authors to postulate that the G4 becomes quartered inside a cavity of the CMG helicase^19^. Batra et al. recently utilized cryo-electron microscopy (EM) analysis to reveal that a single G4 encountered by the replisome lodges into the central channel of CMG helicase in its fully folded conformation, directly impeding replisome progression by disrupting the CMG translocation cycle^37^ (Fig. 1). This work suggests that G4s may be sequestered away from G4 resolvases, raising intriguing questions about fork restart and bypass mechanisms following replisome encounters with these structures. Notably, in Xenopus egg extracts, CMG bypass of large replication obstacles requires auxiliary proteins, such as the G4-resolving helicase DHX36 which allows the approach of leading strand synthesis; the G4-resolving helicase FANCJ enables DNA synthesis past the G-quadruplex^19^. Regulator of Telomere Length 1 (RTEL1), implicated in CMG bypass of DNA-protein crosslinks^38^, plays an important role in mammalian telomeric stability through its action on G4 DNA structures^20^, prompting further interest in its involvement in G4 bypass on the leading or lagging strands.

In addition to genomic G4s encountered by the replicative CMG helicase, a single G4 formed from ssDNA exposed by CMG unwinding has been shown to stall polymerase epsilon in vitro, inhibiting leading strand replicative DNA synthesis and inducing helicase-polymerase uncoupling^39^. This effect was intensified by an increased number of G4-forming sequences and elevated G4 thermal stability. Moreover, G4s formed from ssDNA template exposed in the wake of the MCM helicase ahead of polymerase epsilon, as detected in cells using SM imaging, induce local DSB formation and impede binding of Replication Protein A (RPA), a vital factor in the replication stress response^40^. Consistent with these findings, stabilization of G4 structures by treatment with the G4 ligand telomestatin (TMS), a potent telomerase inhibitor^41,42^, has been shown to induce replication-dependent DSBs, hypermutation, and chromosomal translocations at G4-forming sequences in human cells^43^. Collectively, these studies underscore the critical importance of G4 resolvases and remodeling factors in ensuring smooth fork progression and preventing genomic instability resulting from G4-induced replication stress.

The subsequent sections focus largely on genes mutated in rare diseases that function to overcome the impediment of G4 to nuclear DNA replication; however, mitochondrial G4 DNA is a source of mutagenesis as well^44–46^. Mitochondrial dysfunction is recognized as one of the hallmarks of aging^47^, prompting interest in the relationship of G4 to mitochondrial deletions, point mutagenesis, and loss during aging. Although RNA-G4 metabolism is of great interest in genome biology and human disease^2,48–50^, this expanding field is beyond the scope of the current review which is focused on G4 DNA-induced replication stress.

## DNA translocases: candidates to remodel replication forks stalled by G4 structures

There is mounting evidence that replication fork stalling in eukaryotic cells, triggered by DNA lesions or growth conditions, induces fork structure adjustment and dormant origin firing in response to the replication stress^51^. Replication fork regression, i.e., formation of the branch-migrating four-armed chicken-foot DNA structure similar to the homologous recombination (HR) intermediate Holliday Junction (HJ), is a classic fork remodeling event induced by agents or cellular events that stall DNA synthesis. Fork regression serves to stabilize the replication fork and allows time for signal transduction pathways and a suitable DNA damage/stalled fork response to enable fork restoration, restart, and timely progression of the replisome to complete genomic DNA synthesis prior to cell division. Fork remodeling induced by fork stalling is triggered by a variety of pharmacological compounds, including alkylating agents that induce bulky base damage, HU that interferes with nucleotide pool homeostasis that is essential for polynucleotide synthesis, topoisomerase inhibitors that form covalent protein-DNA complexes, and other drugs that impede fork progression directly or indirectly^52^. Surprisingly, to our knowledge, it is yet to be definitively shown that fork regression is triggered by G4 stabilization at actively progressing replication forks. We anticipate that advances in EM visualization of cellular replication intermediates and protein components of the replisome as well as the development of more sophisticated live cell DNA fiber experimental approaches will lead to an improved understanding of the factors and events that influence fork remodeling. Moreover, structural studies used to characterize mechanistic aspects of G4-induced stalling of the eukaryotic replisome, such as those elegantly revealed by the findings of Batra et al. in the recent *Science* paper^37^, should prove to be informative.

For fork regression to occur upon DNA synthesis slow-down, ATPase-driven DNA translocases play a key role to promote the annealing of nascently synthesized leading and lagging strands at the stalled replication fork. In one prominent mechanism, a DNA translocase acts to unwind the lagging strand DNA duplex, allowing the nascently synthesized lagging strand to become available for annealing of the newly manufactured leading strand and subsequent lagging strand template-directed DNA synthesis that can span up to 1000 nucleotides or more (for review, see ref. ^53^). While it remains to be shown if such DNA translocase-driven fork remodeling can be induced at a replication fork stalled by a G-quadruplex, the idea is certainly plausible and even seems likely. As discussed below, the Helicase-like transcription factor (HLTF) DNA translocase has been implicated in G4 resolution at stalled forks^53^, but its additional role in G4-induced fork regression remains to be demonstrated. Nonetheless, the positioning of HLTF at G4-stalled forks suggests that other DNA fork remodelers may play a role in either G4 resolution or G4-induced fork regression. In addition to HLTF, prominent DNA translocases acting at stalled forks include SWI/SNF Related Matrix-Associated Actin Dependent Regulator of Chromatin Sub-Family A-Like 1 (SMARCAL1) and Zinc Finger RANBP2-Type Containing 3 (ZRANB3). Bi-allelic mutations in *SMARCAL1* are linked to Schimke immuno-osseous dysplasia, characterized by skeletal abnormalities, kidney disease, and a compromised immune system^54^. For ZRANB3, a genome-wide association study of sub-Saharan Africans comprising 18 million autosomal single-nucleotide polymorphisms in >5000 persons from Nigeria, Ghana, and Kenya identified *ZRANB3* as a significant locus for type 2 diabetes^55^. The same research team employed a zebrafish ZRANB3 ortholog model to show that ZRANB3 genetic deficiency resulted in impaired insulin secretion induced by a high glucose condition and reduced pancreatic β-cells due to islet apoptosis^55^, consistent with a working model that ZRANB3 plays an important role in β-cell mass in zebrafish, and perhaps other eukaryotic vertebrates.

## Specialized role of helicase-like transcription factor (HLTF) DNA translocase in the response to G4-induced replication stress

A specific germline missense (E259K) mutation in the *HLTF* gene was identified in familial myelodysplastic syndrome (MDS), a stem-cell disorder characterized by blood cytopenia, bone marrow dysplasia, and high risk of progression to acute myeloid leukemia^56^ (Table 1). The HLTF E259K mutation was found to impair the interaction of HLTF with specific ubiquitin-conjugating enzymes, resulting in impaired polyubiquitination of PCNA and a poor response to agents that induce DNA damage^56^. Other HLTF mutations are associated with cancer progression and reduced patient survival in colorectal cancer and hepatocellular carcinoma^57^.

Several studies have characterized HLTF as a double-stranded DNA (dsDNA) translocase that acts to promote replication fork regression, fork remodeling, and DNA damage tolerance^58^. Most recently, the Cimprich lab characterized HLTF’s role in G4 metabolism. HLTF was shown to resolve DNA-G4s using its ATP-dependent translocase activity. In human cells, HLTF was observed to act in synergy with the mismatch repair factor MSH2 to suppress alternative lengthening of telomeres (ALT), a pathway activated by G4 ligand-induced stabilization, hence promoting telomere maintenance. In response to G4 stabilization, HLTF restrains DNA synthesis, thereby suppressing the replicative bypass of G4 obstacles and consequent mutagenic events. Importantly, loss of HLTF induces G4 accumulation, replication stress, and sensitivity to G4-stabilizing drugs^59^.

## G4-resolving RecQ helicases

**Fig. 2:**
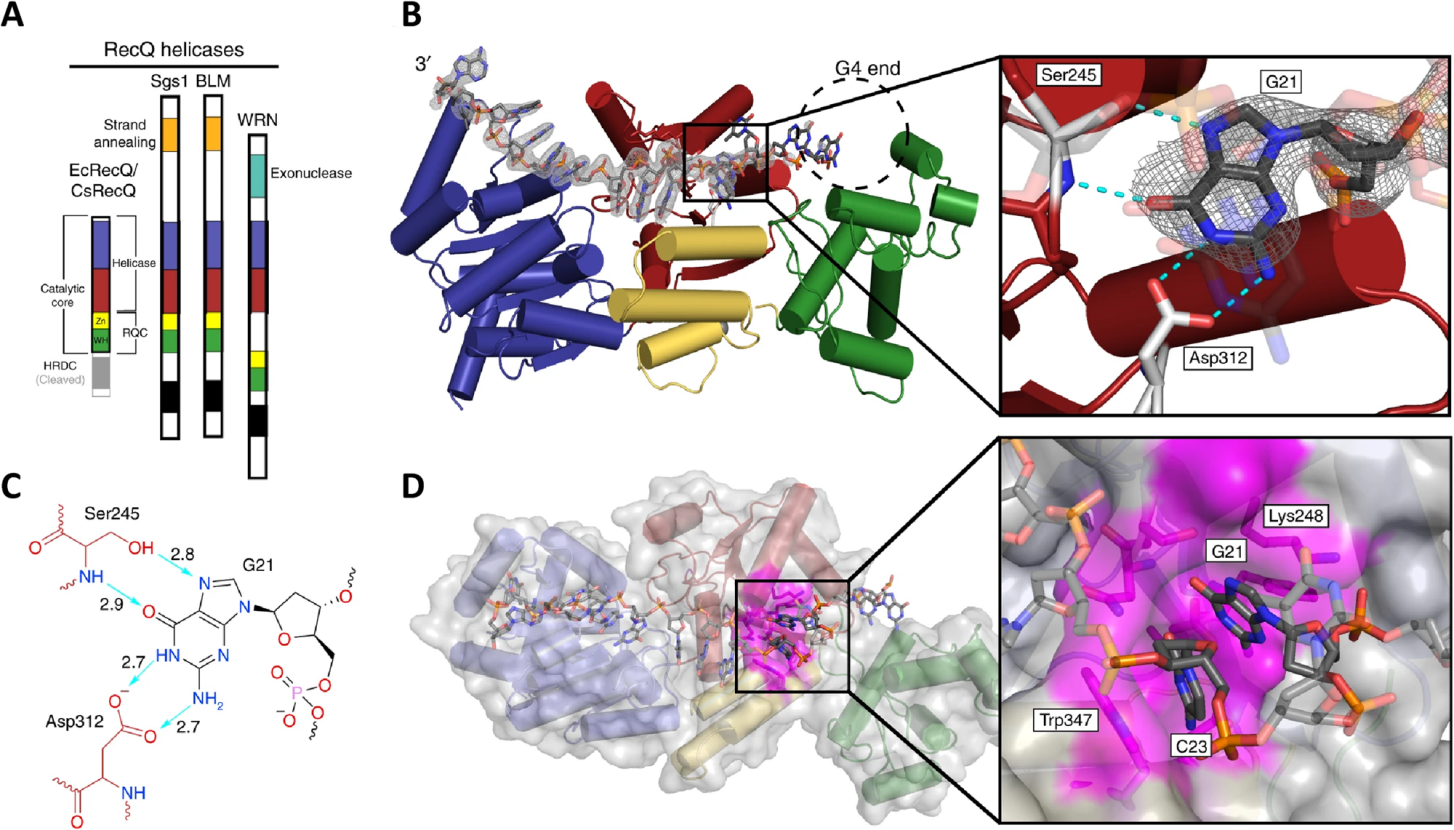
The guanine-specific pocket of the CsRecQ helicase. **A** Domain schematic representation of RecQ helicase family. RecQ comprises two RecA-like helicase folds (blue and red) and a C-terminal domain (RQC). The RQC contains a Zn^2+^-binding domain (Zn, yellow), a winged-helix domain (WH, green) and a helicase and RNaseD C-terminal domain (HRDC, gray). The HRDC has been removed in the RecQ catalytic core. **B** Crystal structure of CsRecQ bound to resolved G4 DNA. Domain colors correspond to **A**. *F*_o_ – *F*_c_ omit electron density contoured at 2.0*σ* is shown. The expected location of the G4 is highlighted. (Insert) The GSP in RecQ binds the flipped guanine with high specificity. Hydrogen bonds are represented by dashed lines. **C** Ligand interaction diagram of the GSP/guanine interface. Bond distances in Å are shown for the hydrogen bonds (teal). Residues from RecQ are red while the guanine is in black. **D** Surface representation of the CsRecQ bound to the resolved G4 with the GSP colored in magenta. (Insert) The flipped G21 is stabilized by hydrophobic interactions and base stacking with C23. From Voter et al., *Nature Communications* (2018) 9: 4201. Ref. ^64^. The work available here: https://www.nature.com/articles/s41467-018-06751-8#rightslink is licensed under the Creative Commons Attribution 4.0 International License.

Helicases are molecular motor enzymes that catalytically disrupt the many hydrogen bonds between bases of various configurations (*e.g*., uni-molecular stem-loop, intermolecular duplex, triplex, G-quadruplex) to alter the structure of the DNA molecule, driven by the chemical energy of nucleoside triphosphate hydrolysis^60,61^. Bi-allelic mutations in human genes encoding four of the five different DNA helicases of the sequence-related RECQ family, named after the prototype *Escherichia coli* RecQ helicase, are linked to distinct diseases characterized by an array of chromosomal abnormalities, accelerated aging, predisposition to cancer, and in some cases, congenital defects (for review, see refs. ^62,63^). Biochemical, genetic, and cell biological studies have provided experimental evidence that certain RECQ helicases implicated in rare genetic disorders resolve G-quadruplexes to preserve genome homeostasis (Table 1), which will be discussed below. A seminal paper from the Keck lab in 2018 revealed the structure of a bacterial RecQ helicase bound to a resolved G4 DNA molecule, elucidating a guanine-specific binding pocket^64^ (Fig. 2). This structure, combined with biochemical data, enabled the authors to propose a guanine-flipping and sequestration mechanism for G4 resolution by the bacterial RecQ helicase; however, the field still awaits new structures of eukaryotic RecQ G4-resolving helicases, including the human representatives implicated in rare diseases. While experimental evidence exists for base flipping by a number of DNA repair and DNA editing enzymes, the mechanism is largely implicated for enzymes that act upon more conventional dsDNA characterized by hydrogen bonding between complementary guanine-cytosine and adenine-thymine base pairs^65^. This hydrogen bonding between complementary bases of double-stranded DNA is quite distinct from the Hoogsteen hydrogen bonding characteristic of the interactions of guanines within a planar quartet array of the G-quadruplex structure. Therefore, it seems reasonable that the guanine flipping/sequestration mechanism proposed for RecQ’s action on G4 DNA offers an evolutionary advantage to achieve the energetic state favorable for G4 resolution.

## Bloom syndrome (BLM)

Mutations in the *BLM* gene are responsible for Bloom syndrome (BS), a rare autosomal recessive disorder characterized by genomic instability, growth deficiency (dwarfism), immunodeficiency, and a markedly increased risk of cancer^66^ (Table 1). Accordingly, BLM-deficient cells fail to properly respond to and repair DNA damage, leading to chromosomal instability. BLM-deficient cells are highly sensitive to DNA-damaging agents and show high levels of sister chromatid exchange (SCE) and spontaneous mutations due to impaired replication fork restart and defective regulation of HR^67–69^. Wu and Hickson provided the first evidence that BLM helicase is responsible for suppressing crossover of chromatid arms during HR^70^. Notably, the Lansdorp group applied single-cell DNA template strand sequencing to map SCEs in BS cells to G4 motifs in transcribed genes^71^, suggesting that BLM limits hyper-recombination at G4 structures found in actively transcribed genes, but the G4-coupled mechanism of BLM action remains to be fully understood.

In vitro, recombinant BLM helicase unwinds or branch-migrates a wide range of DNA structures beyond conventional partial duplex DNA, including HJs^72^ and displacement (D)-loops^73^ that represent intermediates of HR-mediated DSB repair. A collaborative effort between the labs of Hickson and Maizels first demonstrated from bulk measurements that purified recombinant BLM preferentially resolves intermolecular G4 DNA substrates flanked by a 3’ single-stranded tail in an ATP hydrolysis-dependent manner^74^. A subsequent study using a SM Fluorescence Resonance Energy Transfer (FRET) assay demonstrated that BLM unfolds entropically favored intramolecular G4 DNA substrates, such as one formed by the human telomeric sequence^75^. Based on SM FRET assays to measure G4 unfolding by recombinant BLM protein, Wu et al. proposed that the mechanism of BLM action on G4 may be dictated by the molecular architecture of the G4 it encounters^76^, a theme that was proposed for Iron-Sulfur (Fe-S) cluster DNA helicases implicated in other rare hereditary diseases^77^ (see below).

A seminal study placing BLM at the heart of telomere maintenance was conducted by Barefield and Karlseder^78^, in which they demonstrated using fluorescence in situ hybridization with a probe against a telomeric sequence, that metaphase chromosomes of BS fibroblasts display an absence of fluorescence signal at chromosome arms of one (sister telomere loss) or both (telomere-free ends) sister chromatids. BLM was observed to localize at telomeres and coat ultra-fine bridges at telomeres and elsewhere in cells experiencing replication stress induced by aphidicolin exposure. In other studies, BLM was found to interact with several components of the shelterin complex, including POT1, TRF1, and TRF2^79–81^. Single-molecule analysis of replicated DNA (SMARD) in BLM-deficient cells demonstrated a reduced rate of leading strand synthesis using the G-rich template of telomeres, exacerbated by cellular exposure to a G4-stabilizing ligand which caused elevated telomeric G4 detected by the BG4 antibody^82^. These studies collectively mounted a strong case for BLM’s direct role to resolve G4s, especially at telomeres.

## Rothmund–Thomson syndrome/RAPADILINO/Baller–Gerold syndrome (RECQL4)

Mutations in the *RECQL4* gene are linked to three distinct autosomal recessive disorders—Rothmund–Thomson syndrome (RTS)^83^; RAdial hypo-/aplasia, PAtellae hypo-/aplasia and cleft or highly arched PAlate, DIarrhoea and DIslocated joints, LIttle size (height—2 SD or smaller) and LImb malformation, NOse slender and NOrmal intelligence (RAPADILINO) syndrome^84^; and Baller–Gerold syndrome (BGS)^85^—all of which share clinical features of growth retardation and radial defects (Table 1). These pathogenic mutations disrupt RECQL4’s essential roles in DNA replication and repair, mitochondrial maintenance, and telomere stability, resulting in genomic instability and diverse clinical manifestations observed in these syndromes^86,87^.

In addition to its conserved ATPase/helicase core domain found in the other RECQ helicases, RECQL4 features an N-terminal region sharing a unique sequence homology among known human proteins with the yeast DNA replication factor Sld2^88,89^. Notably, experimental evidence suggests a role of eukaryotic RECQL4 in replication initiation^90^. Indeed, RECQL4 interacts with or helps to recruit critical pre-replicative complex factors to origins of DNA synthesis in human cells^91^. This is interesting from the G4 perspective because guanine-rich sequences that are prone to form G4s are found at DNA replication origins and implicated in the initiation of replicative DNA synthesis^92^. Biochemical studies demonstrated that the disordered amino-terminal region of RECQL4 binds tightly to G4 DNA^93,94^, but the regulatory role of such high affinity G4 binding by RECQL4 was elusive. However, the RECQL4-G4 interaction has been reinterpreted through more recent studies^95^. Using a highly sensitive FRET assay with a double-labeled fluorometric substrate consisting of human telomeric sequence that forms G4, Li et al. demonstrated that purified recombinant RECQL4 efficiently resolves the telomeric G4 substrate^95^. Moreover, they determined that RECQL4 interacts with human CST (CTC1–STN1–TEN1), a complex that binds ssDNA, facilitates rescues of stalled replication forks, and promotes telomere replication. Loss of RECQL4 led to accumulation of G4s within the telomeres, thereby resulting in chromosome end dysfunction. A collaborative role of RECQL4 with CST in telomeric G4 metabolism was further demonstrated by single and double knockdown experiments, suggesting coordinate action to promote telomere protection.

## RECON syndrome (RECQL1)

The most recent RECQ helicase disorder to be discovered is designated RECON syndrome, resulting from bi-allelic mutations in the *RECQL1* gene^96^, encoding a DNA helicase with multiple designations in the literature, *i.e*., RECQL1, RECQL, or RECQ1. With only a very limited number of individuals identified to have RECON syndrome, the distinguishing clinical features include progeroid-like facial abnormality, dry eyes, delayed eruption of permanent teeth, skin photosensitivity and xeroderma, and growth retardation (for review, see ref. ^97^). RECQL1-deficient cells display chromosomal instability and a compromised ability to respond to and repair DNA damage induced by topoisomerase inhibitors that introduce replication stress^96^. Prior to its linkage to a rare genetic disorder, cell-based studies garnered evidence for a role of RECQL1 in replication fork restart after exposure of human cells to the topoisomerase I inhibitor camptothecin (CPT)^98^. Biochemical studies had shown that purified recombinant RECQL1 can act upon an array of DNA replication and repair intermediates via its helicase or branch-migration activity^99,100^.

The first evidence that RECQL1 plays a role in G4 metabolism was provided by the Sharma lab, who conducted a transcriptome analysis of RECQL1-depleted human cells revealing that RECQL1 regulates expression of genes that contain predicted G4 motifs in their promoter regions^101^. Furthermore, RECQL1 binding to G4 motifs in the promoters of its target genes was validated by ChIP-qPCR. These findings have prompted interest in RECQL1’s role in transcriptional regulation relevant to cancer and human disease.

Biochemical ensemble experiments with classic radiolabeled intermolecular G4 DNA substrates indicated that purified recombinant human RECQL1 lacked appreciable G4 resolvase activity in vitro under conditions that it was active on conventional partial duplex DNA substrates^102,103^. However, a study from the Xi lab reported that endogenous *Bos taurus* (Bt)RECQL unwound an intramolecular G4 DNA substrate using a FRET-based assay^104^. In a subsequent study, Song et al. used a stopped-flow FRET assay to show that purified recombinant human RECQL1 also unwound an intramolecular G4 DNA substrate. In this study, the authors solved crystal structures of human RECQL1 and BtRECQL in complex with a G4 structure derived from a G-rich *c-Myc* promoter sequence^105^, leading them to propose a model for helicase engagement whereby coordination of G4 recognition and unwinding operates in manner similar to that proposed for duplex DNA unwinding by RECQL1^106^. The relationship of RECQL1’s role in the replication stress response to its biochemical interaction with G4 and contribution to the molecular pathology of disease remains to be established and necessitates future study.

## Werner syndrome (WRN)

Werner syndrome (WS) is a classic premature aging disorder caused by bi-allelic mutations in the *WRN* gene, encoding the WRN helicase-exonuclease^107^ (Table 1). Cells from WS patients exhibit telomere dysfunction and loss, genomic instability in the form of chromosomal breaks, rearrangements, deletions, and cellular senescence^108–110^. Biochemical and cell-based studies have suggested an important role of WRN in G4 metabolism. Fry and Loeb were the first to report that purified recombinant WRN resolves G4 DNA structures, including a DNA substrate containing CGG repeats implicated in Fragile X syndrome^111^. In a subsequent study, they reported that WRN collaborates with the replicative DNA polymerase delta to catalyze efficient DNA synthesis past a G4 obstacle in vitro^112^. Additional studies have confirmed WRN’s catalytic resolution of various G4 DNA substrates^113,114^. Cell-based studies implicate a partnership of WRN with the ssDNA binding protein RPA, induced by casein kinase 2 (CK2) WRN phosphorylation, to promote replication fork restart under conditions of G4-induced replication stress^115^. Knockdown of the E3 ubiquitin ligase HERC2, which modulates this WRN-RPA interaction^116^, reduced the interaction of WRN (and BLM) with RPA. Cells expressing ubiquitin ligase-dead HERC2 also display elevated nuclear G4 levels, suggesting HERC2’s enzymatic activity is required for proper G4 processing by the WRN-RPA complex^116^.

WRN also associates with Werner Interacting Protein 1 (WRNIP1) and genetically interacts with Breast Cancer Gene 2 (BRCA2), two proteins that contribute to replication fork stabilization under conditions of stalled DNA synthesis. WRNIP1 depletion results in G4 accumulation and chromosomal aberrations following treatment with G4 ligands^117^, suggesting a potential collaborative interaction with WRN for G4 resistance; however, WRN’s interactive role with WRNIP1 was not investigated in this study. In BRCA2-deficient cells, WRN protects stalled replication forks from excessive degradation during nucleotide pool depletion caused by HU exposure^118^, raising the possibility that G4-induced replisome stalling might also elicit a WRN-dependent mechanism to preserve genomic stability when BRCA2 is mutated in proliferating cancer cells with high rates of DNA synthesis and elevated replication stress. Further studies are warranted to address this hypothesis, especially given that WRN and BRCA2 have roles in telomere metabolism (see below).

Cells lacking functional WRN, or expressing a helicase-dead WRN mutant, display deletion or loss of telomeres from single sister chromatids, a phenotype termed sister telomere loss (STL)^109^. WRN’s activity is specifically required to resolve replication challenges unique to synthesis on the telomeric lagging strand, which uses the G-rich strand as a template^109^. While exogenous full-length WRN can recover the increase in STL on the lagging strand in WS fibroblasts, helicase-dead WRN cannot. This points to a possible role for WRN in resolving lagging strand template G4s in telomeres by enabling processive DNA synthesis and suppressing the degradation of single-stranded telomeric DNA. WRN was further implicated in telomere maintenance by shuttle vector (SV) mutagenesis assays performed in telomerase-negative U2OS cells that maintain telomere length through the ALT pathway^119^. Depletion of WRN dramatically elevates mutation frequency in SVs containing telomeric repeats [TTAGGG]_6_ compared to a control vector lacking G4-forming sequences. This elevated mutation frequency in WRN-deficient cells is primarily due to a significant rise in large sequence deletions and rearrangements, consistent with a model in which WRN plays a specialized role in resolving telomeric G4s. It would be of interest in these cell-based assays, which assess the importance of WRN in telomeric DNA synthesis of the G4-forming strand, to determine whether this effect is exacerbated by treatment with a G4-stabilizing ligand or co-deficiency of other G4-resolving helicases also implicated in telomere metabolism (e.g., BLM). Moreover, the demonstration of genomic telomeric deletion in the ALT pathway cells was not examined. Consistent with a role of WRN at telomeres, premature aging phenotypes in late generation telomerase-deficient *(Terc-/-)* mice are observed with co-deficiency of WRN^120^; however, to our knowledge, the precise molecular function(s) of WRN in telomeric G4 homeostasis in *Terc-/-* mice has not been definitively characterized.

WRN was found to interact with the shelterin protein Telomeric Repeat-binding Factor 2 (TRF2)^121^; however, to our knowledge, a functional interaction of WRN with TRF2 in the context of telomeric G4 DNA has not been reported. WRN also interacts with FEN1^122–125^, a protein implicated in Okazaki fragment processing during semi-conservative DNA replication in eukaryotic cells^126^. Moreover, interactions of FEN1 with WRN and TRF2 were shown to be required to prevent lagging strand STL^127^. An intriguing hypothesis is that WRN (and/or BLM, which also interacts physically and functionally with FEN1 to cleave unstructured 5′ flap structures^128^) might facilitate secondary structure resolution (*e.g*., G4) within 5′ flaps to allow for efficient FEN1 tracking to the flap junctions that serve as substrates for FEN1 cleavage. Indeed, a (CGG)_6_ repeat sequence implicated in the trinucleotide repeat neurodegenerative disorder Fragile X syndrome^129^, which forms a non-canonical DNA structure centrally positioned in the 5′ flap DNA substrate, inhibits 5′ flap cleavage by purified recombinant human FEN1^130^. To our knowledge, the ability of WRN or BLM to stimulate FEN1 cleavage of 5′ flaps possessing G4 structures has not been addressed. Given the requirement of WRN to facilitate lagging strand synthesis at telomeres^109^, and the importance of WRN’s interaction with FEN1 to process branch-migrating DNA structures associated with stalled replication forks^124^ to preserve telomere stability^127^, further studies of WRN’s role in telomeric G4 DNA processing are warranted.

Beyond its role at specific G-rich-chromosomal loci such as telomeres that form G4s, WRN may have a broader impact on chromatin biology through its involvement in the G4-induced replication stress response. Recently, WRN was found to co-localize with G4s in human dermal fibroblasts (HDFs) exposed to pyridostatin (PDS) or depleted of BMI1, a Polycomb complex factor implicated in histone modification and chromatin compaction^131^. Acute loss of BMI1 caused heterochromatin relaxation and induced G4 formation that co-localized with genomic DNA damage. WRN depletion also elevated G4 abundance, and HDFs from individuals with WS, or the genetically inherited Hutchinson-Gilford progeria syndrome linked to mutations in the *LMNA* gene encoding a nuclear membrane protein^132,133^, displayed heterochromatin loss and nuclear envelope anomalies characteristic of BMI-depleted HDFs. Hanna et al. proposed that G4 suppression via heterochromatin preservation is important for cells to mitigate replication stress, a process that is compromised in premature aging syndromes^131^. These findings likely relate to an earlier study in which Zhang et al. elucidated a role of WRN to maintain heterochromatin integrity in a WS model of aging stem cells; moreover, an examination of primary dental pulp mesenchymal stem cells of old individuals (58-72) demonstrated a concomitant reduction in heterochromatin marks and WRN protein, suggesting a WRN-dependent pathway of physiological aging^134^.

## Telomeric G4 transactions of breast cancer gene 2 (BRCA2)/Fanconi anemia group D1 (FANCD1)

As mentioned in the previous section, WRN plays a prominent role in telomere maintenance and genetically interacts with BRCA2, also known as FANCD1, to protect forks under conditions of replication stress. Although BRCA2 is not a DNA helicase, it acts as a mediator by facilitating the loading of RAD51 onto single-stranded DNA (ssDNA) at stalled forks, stabilizing RAD51 filaments and promoting homologous recombinational repair^135,136^. Mutations in the *BRCA2* gene result in significantly increased susceptibility to breast and ovarian cancers^137^, and moderately increased susceptibility to other cancers such as colorectal^138^, prostate^139^, or pancreatic^140^. Additionally, bi-allelic mutations in *BRCA2* are associated with Fanconi Anemia (FA) (complementation group D1 (FA-D1)), a rare hereditary disorder characterized by progressive pancytopenia with bone marrow failure, congenital malformations, and predisposition to malignancies^141–143^ (Table 1).

The aforementioned stabilization of RAD51 on resected DNA by BRCA2 protects the replication fork from exonuclease-driven degradation. Loss of BRCA2 results in unrestrained fork resection and subsequent chromosomal aberrations, driving tumorigenesis^144^. BRCA2 localizes to a sub-population of telomeres during S-phase, and its deficiency induces telomere loss, leading Lee et al. to investigate if BRCA2 transactionally affects human telomeric DNA structures^145^. They demonstrated that BRCA2 dynamically interacts with telomeric G4s and their G-triplex (G3) intermediates during DNA replication. This binding enables RAD51 loading onto telomere ssDNA, prompting fork restart. BRCA2 also suppresses MRE11-mediated resection of G4-stalled forks, preventing telomere fragility and shortening^145^, consistent with earlier observations that BRCA2 deficiency causes the accumulation of common fragile sites, interestingly enriched in the G-rich lagging strand, and elevated telomere SCE^146^. BRCA2 deficiency sensitizes mouse embryonic fibroblasts (MEFs) to the G4 ligand PDS, resulting in a significantly higher percentage of fragile telomeres^147^. In human cancer cells, combined BRCA2 depletion and PDS treatment induced telomeric DNA damage and micronuclei formation due to interference with G4 dynamics and accumulation of unresolved R-loops^148^.

Considering the role of BRCA2 in G4 metabolism, several studies have identified G4 DNA as a potential therapeutic target in BRCA2-deficient cancer cells^149–151^. Some G4 ligands have also been tested in clinical trials in BRCA2 mutated cancers. Specifically, the G4-stabilizer CX-5461 (Pidnarulex) was tested in a phase 1 clinical trial^152,153^. Reportedly, of the 29 patients with germline or somatic mutations in DNA repair genes including *BRCA1*, *BRCA2*, *PALB2*, and *TP53*, four exhibited partial response in which tumor size was decreased, four exhibited durable stable response in which tumor size did not increase for more than six months, and seven exhibited stable response in which tumor size did not increase for less than six months. G4-stabilizing drugs continue to be attractive candidates for anti-cancer drug treatment of patients with DNA repair-deficient tumors.

In recent work by the Smogorzewska lab and collaborators, G4 structures were implicated as a target for anti-cancer therapies against *BRCA2-*deficient granule cell progenitors (GCPs), leading to medulloblastoma (MB), based on the sensitivity of replication speed in GCPs exposed to the G4 ligand PDS^154^. Moreover, knockout of the gene encoding G4-resolving helicase PIF1 in primary MB tumor cells caused elevated PDS-induced genomic instability. In a subsequent section, we expand upon the additional evidence for the role of PIF1 in G4 DNA metabolism.

## G4-resolving Fe-S cluster helicases

Fe-S cluster helicases represent a family of conserved prokaryotic and eukaryotic helicases that possess not only the two conserved RecA-like motor domains shared by superfamily 2 helicases (including the RecQ family) but also the Fe-chelating cluster marked by conserved cysteine residues, and a structural Arch domain, both residing within the conserved ATPase/helicase core domain^63^. It is remarkable that the crystal structures of three Fe-S cluster XPD helicases were published within six months of each other, revealing tremendous structure-function insights and the molecular basis for helicase inactivation and rare disease phenotypes^155–157^. Importantly, the Fe-S cluster helicases all unwind conventional duplex DNA substrates with a 5′ to 3′ directionality, opposite to the 3′ to 5′ directionality of RecQ helicases^63^. The Fe-S helicases play prominently in human disease and G4 transactions (Table 1), as will be highlighted in the subsequent sections.

## Fanconi anemia group J (FANCJ)

Mono-allelic mutations in the *FANCJ* gene increase susceptibility to multiple types of cancer, including hereditary breast and ovarian cancer, melanoma, prostate cancer, and colon cancer^158^. Bi-allelic *FANCJ* mutations are linked to the rare genetic disorder Fanconi anemia (FA), characterized by chromosome instability, bone marrow failure, predisposition to blood cancers, and hypersensitivity to chemotherapeutics^159–161^ (Table 1). FA is complex and heterogeneous, with currently 23 genes linked to the chromosomal instability disorder (Fanconi Cancer Foundation). Failure to effectively repair DNA damage, particularly interstrand cross-links (ICLs), and respond to replication stress, are generally believed to be the primary cause underlying the FA mutant phenotypes^162^. Currently, the only treatment to restore hematological function in FA patients is bone marrow transplantation, whereas cancer treatment in FA patients requires surgical resection due to the hypersensitivity of patients to radiotherapy or chemotherapy^163^.

FANCJ, also known as BRIP1 or BACH1, plays a crucial role in the repair of ICL-generated DNA DSBs by HR^159–161,164^ that is mediated in part by its interaction with the mismatch repair complex MutLα^165^. FANCJ also repairs DNA damage that forms independently of ICLs by associating with other proteins such as BRCA1^166^ and MRE11^167^. In addition to ICL and DSB repair, FANCJ is an important player in the replication stress response (for review, see refs. ^168,169^). FANCJ is found at active replication forks, where it influences the status of other replisome proteins and promotes fork integrity in coordination with HLTF^170^. FANCJ’s partnerships are likely germane to observations that FANCJ influences unrestrained replication and ssDNA gap formation under conditions of cellular exposure to a poly(ADP-ribose) polymerase inhibitor (PARPi)^171^. The crosstalk of FANCJ with PARP1 during cellular DNA replication is complex, as it was recently shown that FANCJ enhances PARP1 PARylation in BRCA1-deficient cells^172^.

Collective experimental evidence from multiple labs makes a strong case that FANCJ is a prominent helicase to resolve G4s in vivo (for review, see ref. ^168^). The Lansdorp lab determined that the *dog-1* helicase in *Caenorhabditis elegans*, sharing sequence homology to FANCJ, can suppress germline and somatic DNA deletions initiated at G-rich sequences predicted to form G-quadruplexes^173^. The deletions were found to reside most prominently at the 3′ end of poly-guanine tracts and to only involve approximately 50% of these tracts, leading to the proposal that the deletions may be a consequence of G4s that arise during lagging-strand DNA replication. We showed that human recombinant FANCJ helicase can resolve G-quadruplex DNA substrates in vitro and characterized the catalytic and substrate requirements^103^. In this study, we demonstrated that acute depletion of FANCJ in cancer cells induces hypersensitivity to the G4-stabilizer TMS, accompanied by increased levels of DNA damage as detected by γH2AX, as well as DNA synthesis inhibition and induction of apoptosis^173^. In a subsequent study, London et al. confirmed that FANCJ resolves G4 DNA substrates in vitro and that a FA-J patient cell line displayed an abundance of large genomic deletions in the vicinity of predicted G4-forming sequences^174^.

Over the years, multiple studies—in part prompted by the development of G4-specific probes and antibodies—have demonstrated that FANCJ deficiency is associated with increased cellular G4 levels, hypersensitivity to G4-stabilizing compounds, and replication stalling at G4s^175–178^. In contrast to the repair of highly destructive ICLs, genetic characterizations of partially defective FA patient-derived FANCJ helicase domain mutants suggest that a minimal threshold of ATP-dependent FANCJ catalytic activity is required to resolve G4s in human cells^179^. The prominent role of FANCJ in G4 resolution appears to be evolutionary conserved, as demonstrated in chicken DT40 bursal lymphoma cells^176^, *C. elegans* gonadal germ cells^180^, and reconstituted Xenopus egg extracts^176^. In mice, experimental evidence suggested that the metabolism of G4 structures is unaffected by loss of FANCJ, as *Fancj-/-* MEFs do not show hypersensitivity to G4-stabilizing compounds^181^. Nonetheless, FANCJ deficiency causes microsatellite instability in both murine^181^ and human^182^ cells, due to the inherent difficulty of replicating these repetitive genomic elements. Moreover, recent evidence shows that FANCJ loss results in extensive repeat expansion in the *FMR1* gene in a mouse model of Fragile X-related disorder, particularly in intestine and male germline^183^. Considering that the *FMR1* gene repeats form G-quadruplexes^184,185^, and that these secondary DNA structures play a role in repeat expansion, this new evidence prompts further studies of FANCJ’s role in the resolution of G4 and other secondary DNA structures in Fragile X syndrome.

**Fig. 3:**
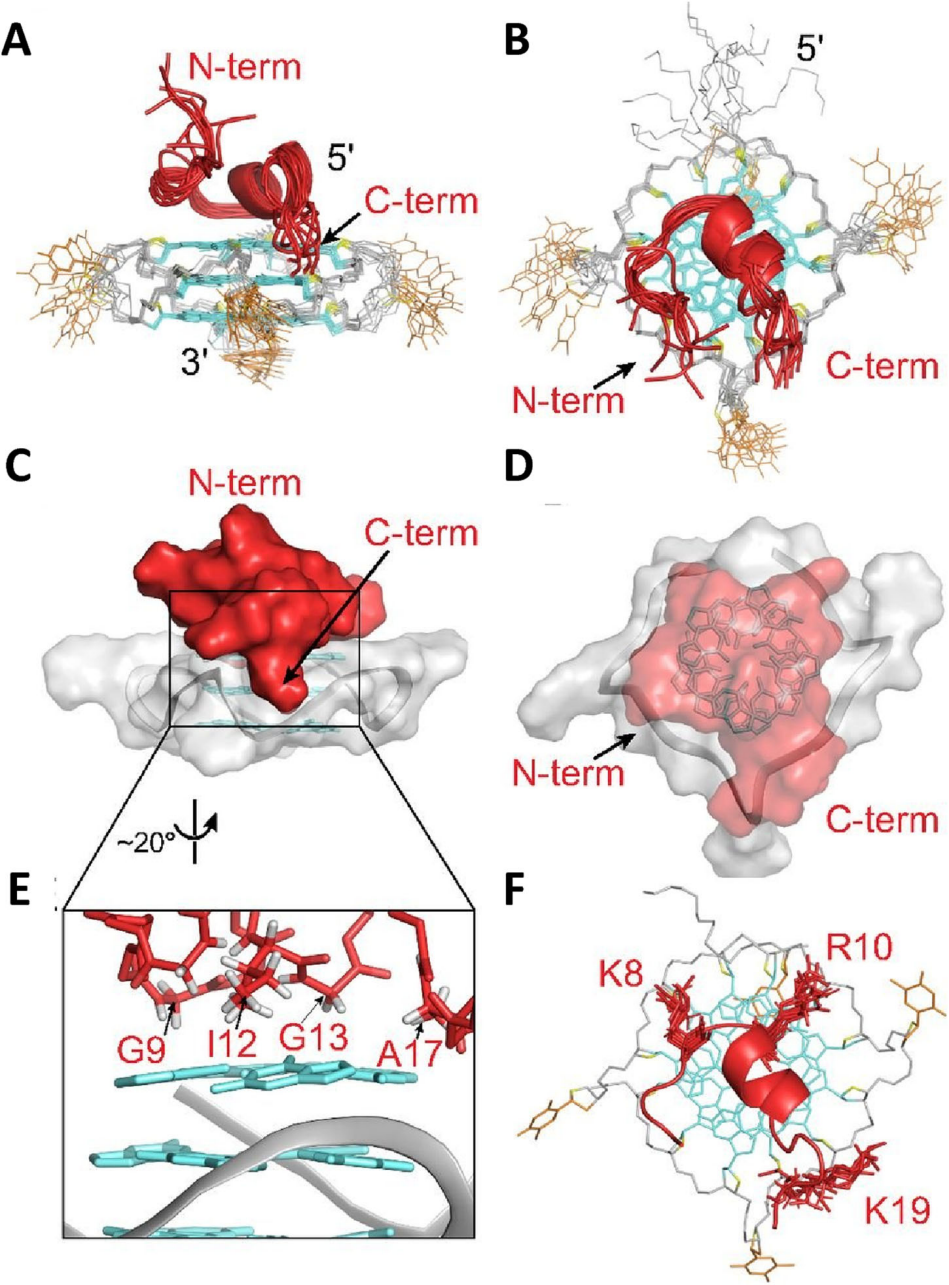
NMR solution structure of Rhau18–T95-2T complex. **A** Side and **B** top view of the 10 superimposed lowest-energy structures. **C** Side- and **D** top-view surface representation of the complex. **E** Details of the intermolecular interactions between peptide residues (G9, I12, G13, and A17) and DNA guanine bases of the 5′-end G-tetrad. **F** Details of the intermolecular interactions between peptide positively charged side-chains of K8, R10, and K19 and the DNA phosphate backbone. Rhau18, red; guanines, cyan; thymines, orange; DNA backbone, gray; O4′ atoms, yellow. From Heddi et al., *Proceedings National Academy Sciences* *U.S.A*. (2015) 112(31) 9608-13. Ref. ^188^. Per the PNAS Executive Editor, because this material was published after 2008, a copyright note is not needed.

Additional studies have elucidated the biochemical properties of FANCJ in G4 resolution. The conserved Fe-S domain of FANCJ, essential for DNA unwinding and ICL repair, also plays a critical role in G4 metabolism, as cancer-associated mutations that disrupt the Fe-S cluster confer cellular sensitivity to G4-stabilizing compounds^186^. Distinct from other G4-resolving helicases, FANCJ harbors a G4 recognition site for precise targeting to G4-forming DNA^187^, which could in part explain its prominent role to resolve G-quadruplexes in vivo. Notably, Wu and Spies determined that a peptide designated as a DHX36-specific motif (DSM) found in the helicase RHAU/DHX36 sequence, previously determined by NMR spectroscopy to form a complex with parallel-stranded G4 DNA^188^ (Fig. 3), shared unique similarity to FANCJ, but not other G4-resolving helicases (e.g., RecQ helicases, Pif1, RTEL1)^187^, suggesting a potentially conserved mechanism of G4 interaction and/or resolution between FANCJ and RHAU/DHX36. However, much remains to be learned about the mechanism of action for resolution or unfolding of multi-stranded and uni-molecular G4 structures by G4 helicases and other G4-interacting proteins. A distinguishing feature of FANCJ is that it efficiently resolves entropically favored uni-molecular G4s in vitro^77^, the most common G4 DNA structure predicted to form in vivo^189^.

The ssDNA binding protein RPA stimulates FANCJ G4 resolution^103^. Mechanistically, G4 unwinding by FANCJ enables RPA loading onto the unwound DNA, hence protecting ssDNA and promoting DNA synthesis past G4s in human cells^40^. Cooperation between FANCJ and RPA is also necessary to recruit specific checkpoint proteins and implement an efficient response to G4-associated replication stress^190^. In chicken DT40 cells, experimental data suggest that FANCJ cooperates with the G4-resolving helicases BLM or WRN (which have the opposite directionality of FANCJ) to promote epigenetic stability. In this context, Rev1, a translesion synthesis polymerase, has also been implicated in assisting replication past such secondary structures. Rev1 appears to act downstream or in parallel to FANCJ and BLM/WRN, providing a mechanism for bypassing unresolved G4s^191^. Crosstalk between FANCJ and BLM helicases was first evidenced by their physical and functional interaction in human cells, as they co-localize to nuclear foci upon replication stress^192,193^. In this work, FANCJ deficiency caused BLM degradation in a proteasome-mediated pathway, suggesting an intimate relationship between the two helicases. In a more recently published study, it was determined by proximity ligation assay that exposure of glioblastoma cells to the G4 ligand RHPS4 strongly increased the FANCJ-BLM interaction, suggesting their direct collaborative role in the response to G4-induced replication stress^194^. RHPS4 treatment caused FANCJ recruitment to telomeres^194^, consistent with previous evidence that FANCJ localizes to telomeres in ALT-positive cells^195^ and FANCJ-deficient cells are hypersensitive to the telomeric G4 ligand TMS^103^. Co-deficiency of FANCJ and BLM induced telomeric aberrations to a level greater than that observed for singly FANCJ- or BLM-deficient cell lines^194^. Altogether, these findings suggest that FANCJ and BLM have collaborative as well as compensatory roles that facilitate replication following G4-induced stress and promote telomere protection.

Other proteins that collaborate with or partly substitute for FANCJ in G4 resolution include DHX36^19^ and RAD52^196^. DHX36 primarily enables the replicative helicase CMG to bypass the G4 blocks present on the DNA leading strand, followed by unwinding of the G4 by FANCJ^19^. DHX36 and FANCJ act together to unwind G‑quadruplex structures formed at R-loops (G‑loops) with DHX36 initiating G4 resolution and FANCJ further processing the structure to enable its complete disassembly. This cooperation triggers nucleolytic incision and subsequent DNA synthesis, preventing pathological accumulation of G4s and R‑loops and thereby protecting genome stability^197^. RAD52 is instead recruited to G4 sites after fork breakage and DSB formation to promote HR and recruit specialized endonucleases for G4 removal^196^. RAD52 exhibits a synthetic lethal interaction with FANCJ, as double-deficient cells show substantially increased DNA damage and cell death after treatment with G4-stabilizing compounds^196^. Like FANCJ, RAD52 was found to promote HR repair. Moreover, RAD52 is proposed to aid in the recruitment of the structure-specific nuclease XPF to cleave and eliminate G4s at the ends of DSBs to facilitate their efficient processing. In addition, RAD52 interacts with the endonuclease MUS81 to cleave G4-induced stalled replication forks to facilitate HR-mediated fork restart. While the role of FANCJ in G4 resolution has clearly been demonstrated in human cells and model systems, its collaborative action with other factors like RAD52 remains to be well characterized.

FANCJ also cooperates with the mismatch repair proteins MutSβ and MutLβ to resolve G-loops, which impede DNA replication. MutSβ specifically binds to G4s, while MutLβ recruits and physically interacts with FANCJ^198^. While FANCJ plays a prominent role in G4 resolution, additional helicases such as DDX17^199^ and others^200^ are involved in R-loop formation and resolution during transcription or CRISPR-mediated gene editing that potentially affect replication or other processes of genome metabolism.

## Regulation of telomere elongation helicase 1 (RTEL1)

Bi-allelic mutations in *RTEL1*, encoding a Fe-S cluster helicase^201^, are linked to the bone-marrow failure disease Dyskeratosis congenita (DC) in which patients present abnormal skin pigmentation, nail dystrophy, oral leukoplakia, and an elevated risk of cancer^202^ (Table 1). *RTEL1* mutations are also implicated in Hoyeraal–Hreidarsson syndrome (HHS), classified as an aggressive variant of DC^203–205^ (Table 1). Clinical features of HHS include bone marrow failure, intrauterine growth retardation, cerebellar hypoplasia, and immunodeficiency. More recently, a homozygous missense mutation in the RTEL1 helicase domain was linked to Infantile-Onset Ulcerative Colitis and Severe Immunodeficiency characterized by a failure to thrive, dysmorphic features, and immunodeficiency^206^.

DC/HHS is a complex heterogeneous disease characterized as a telomere biology disorder that is linked to pathogenic germline mutations in a minimum of 18 different genes, including *RTEL1*^201^. In a key molecular and cellular characterization of RTEL1’s function, Vannier et al. attained experimental data supporting a model in which RTEL1 resolves telomeric D-loops (T-loops) and G-quadruplexes at telomeres to suppress telomeric fragility and loss^20^. Beyond its function(s) in telomere metabolism, RTEL1 is believed to have a broader role in genomic maintenance by its association with the replisome to promote genomic and telomeric replication^207^ and interaction with the SLX4 tumor suppressor which serves as a scaffold in DNA repair and chromosomal stability^208^. Together, RTEL1 and SLX4 are proposed to suppress replication-transcription conflicts and replication defects, even in unstressed cells. RTEL1 interacts with Polymerase delta-interacting protein 3 (POLDIP3), a subunit of DNA polymerase δ and the TREX complex, facilitating localization to chromatin during replication stress induced by cellular exposure to CPT. Depletion of RTEL1 or POLDIP3 causes a mutual reduction in chromatin binding for both proteins, resulting in R-loop accumulation^209^. In other work, RTEL1 was shown to be required for stabilization of common fragile sites and telomeres following replication stress by facilitating mitotic DNA synthesis at G4-associated R-loops^210^. In this study, an SLX4-RTEL1 interaction was also implicated, which the authors determined to facilitate recruitment of other proteins (e.g., RAD52, POLD3) to enable completion of DNA synthesis in under-replicated regions of the genome. Kotsantis et al. reported that transcriptional changes in *Rtel1*-/- cells were mostly observed for genes in which the promoter elements are predicted to form G4s^211^. Removal of R-loops by RNase H1 overexpression alleviated global replication stress, prompting the authors to propose that RTEL1 resolution of G4 DNA in the displaced strand of R-loops helps to avoid replication-transcription conflicts^211^.

## DEAD/H-box helicase 11 (DDX11)

Originally designated ChlR1, DDX11 is a member of the Fe-S helicase family^212^ shown to be required for sister chromatid cohesion in mammalian cells^213^. Bi-allelic mutations in the *DDX11* gene are linked to the rare genetic disorder Warsaw Breakage syndrome (WABS), a disease characterized clinically by microcephaly, growth retardation, and abnormal skin pigmentation^214^ (Table 1). Since it was first identified, numerous *DDX11* mutations have been linked to WABS^215–219^.

WABS patient-derived cells display excessive chromosomal breakage upon exposure to the DNA cross-linking agent mitomycin C (MMC) or the topoisomerase I inhibitor CPT, and exhibit sister chromatid cohesion defects exemplified by the railroad appearance of chromatid pairs in metaphase spreads of mitotic cells^214^. This led to the classification of WABS as a cohesinopathy with a distinct clinical and molecular phenotype. Further evidence links DDX11 with a role in cohesin maintenance as multiple groups have conducted CRISPR screens in targeted-knockout cells and identified genetic interactions of DDX11 with factors involved in sister chromatid cohesion^220–222^. In DT40 cells, it was shown that DDX11 functions in a pathway separate from CTF18, a PCNA clamp loader with a role in sister chromatid cohesion, as loss of both genes caused synthetic lethality. Moreover, depletion of the cohesin-removal factor WAPL suppresses the impaired proliferation and cohesion defects of the *ctf18 ddx11* double mutants, suggesting that cohesin release by WAPL can bypass the complementary roles of the two fork protection factors^223^.

Emerging evidence from biochemical and cell biological studies suggest an important molecular function of DDX11 in the resolution of DNA secondary structures, particularly G4. Studies of DDX11-depleted cells demonstrated their sensitivity to DNA-damaging agents, including G4-stabilizing ligands. The de Lange lab reported sensitivity of both DDX11 knockout cells and WABS patient cells to multiple G4 stabilizers^224^. Jegadesan and Branzei also reported the sensitivity of DDX11 knockout cells to the G4 ligand PDS^225^. Consistent with the data from cell-based experiments, biochemical characterization of purified recombinant DDX11 revealed its ability to bind and resolve G4 DNA^226^. Moreover, DDX11 has a strong substrate preference for an anti-parallel two-stranded substrate G4 compared to a parallel four-stranded G4^226^ or a uni-molecular G4^77^. Human telomeric G4 can assume a variety of intramolecular or intermolecular G4 topologies, including a two-stranded anti-parallel configuration^227^; however, a definitive role of DDX11 in telomeric G4 resolution within cells remains to be seen.

The precise function(s) of DDX11 in G4 DNA metabolism in cells is still unclear. However, work from the Hurwitz lab suggesting DDX11’s involvement in lagging-strand DNA processing may be relevant. DDX11 was found to directly interact with FEN1, a protein that is implicated in Okazaki fragment processing during semi-conservative DNA replication in eukaryotic cells^228^. However, it remains to be shown whether DDX11’s ability to resolve G4 DNA is coordinated with its FEN1 interaction. An intriguing model is that DDX11 contributes to the processing of long structured guanine-rich 5′ flap structures prone to form extensive G4 that would be resistant to FEN1 action. This coordination between DDX11 and FEN1 would facilitate optimal tracking on the ssDNA flap by FEN1 and subsequent cleavage at the elbow of the 5′ flap junction to create a ligatable nick during lagging-strand DNA synthesis.

It seems probable that multiple factors contribute to the recruitment of DDX11 to secondary DNA structures like G4s encountered by the replisome, especially when replication stress is elevated. A prominent factor in this regard is Timeless (Tim), a member of the fork protection complex^229^. The Sale lab and collaborators showed that Tim-deficient DT40 cells display decreased replication through a G4 motif, suggesting its involvement in G4 resolution^230^. A complex of Tim with another fork protection factor known as Tipin likely functions in the recognition of G4 structures as the Tim-Tipin complex binds G4 DNA much more tightly than ssDNA or dsDNA^230^. However, Tim lacks catalytic activity, suggesting it must collaborate with a G4-resolving helicase. DT40 cells co-deficient in Tim and DDX11 displayed a similar decrease in replication through the G4 motif compared to either Tim- or DDX11-knockout cells, suggesting DDX11 and Tim operate in the same pathway to overcome G4 obstacles^230^. Consistent with this model, Tim enhances the ability of DDX11 to bind and resolve a two-stranded anti-parallel G4 DNA substrate in vitro^231^.

**Fig. 4:**
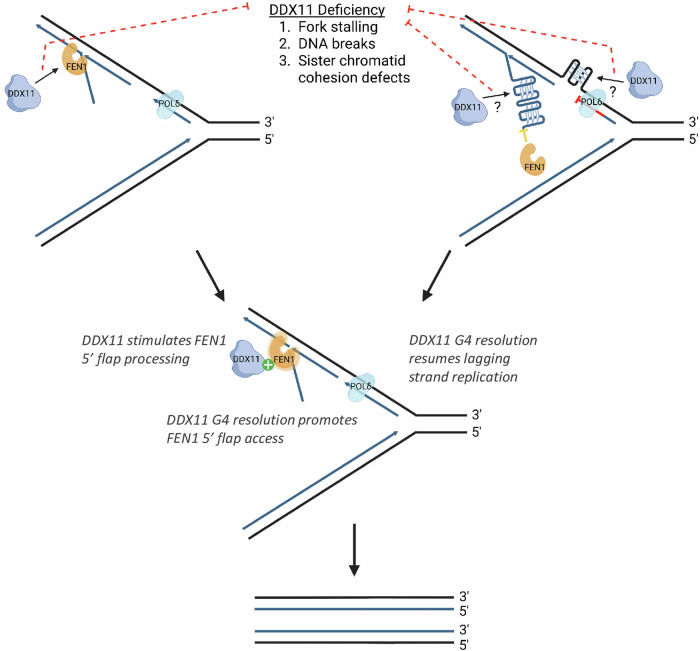
Conceptual dynamics of G4 resolution by DDX11 during DNA synthesis of the lagging strand at stressed replication forks. The role of DDX11 to resolve G4 structures that form from transient ssDNA in the template or nascently synthesized lagging strand and its interaction with FEN1 to stimulate 5′ flap processing may serve to preserve genomic stability during DNA replication. See text for details. Created in BioRender. Herr, L. (2025) https://BioRender.com/lvrqjly.

How DDX11’s involvement in G4 resolution and Okazaki fragment processing relates to its role in suppression of sister chromatid cohesion defects characteristic of WABS has been of interest. Van Schie et al. reported that WABS patient cells treated with G4-stabilizing ligands show an increase in chromosome breaks and sister chromatid cohesion defects^224^. This phenotype can be corrected by overexpression of wild-type DDX11, but not by a DDX11 site-directed helicase-dead mutant, supporting the notion that DDX11-mediated G4 resolution is crucial to prevent fork stalling and promote proper sister chromatid cohesion. Whether DDX11’s role in G4 metabolism in human cells is confined to resolution of G4 in the lagging strand template remains to be seen. Carefully designed DNA fiber studies may help to address the molecular role of DDX11 and associated factors in dealing with G4-induced replication stress. It has been suggested that loss of G4 resolution due to a DDX11 deficiency could contribute to the sister chromatid cohesion defect seen in WABS patient cells through inhibition of cohesin loading by G4 accumulation or interference with removal of cohesin to facilitate repair of DNA breaks caused by fork stalling^224^. The bulky G4 secondary structures may disrupt proper cohesin function, resulting in chromatid cohesion defects. Further, the de Lange group alluded to the potential role of DDX11 in resolution of G4 structures in the lagging strand to allow for efficient replication and maintenance of sister chromatid cohesion^224^. In Fig. 4, we illustrate the conceptual dynamics of DDX11 functions in G4 resolution involving the lagging strand at stressed replication forks.

## Replication fork auxiliary factors with roles in G4 DNA processing

In addition to the RecQ and Fe-S helicases, as well as DNA translocases mentioned earlier, there are several known factors elicited upon G4-induced replication stalling which will be addressed in this section. An ongoing challenge is to delineate the crosstalk among these proteins in G4-centric pathways of genomic DNA metabolism in cells, with a special emphasis placed on the requirement to efficiently resolve G4 structures on the leading or lagging strand template that interfere with replisome progression, smooth DNA synthesis, and coordination of leading and lagging strand replication.

## DNA replication helicase/nuclease 2 (DNA2)

DNA2 is a highly conserved enzyme with both nuclease and helicase activity that is implicated in human diseases^232^ (Table 1). DNA2 participates in multiple processes to maintain genome stability, including processing of Okazaki fragments during lagging-strand DNA synthesis, DNA end resection during DSB repair, and restart of arrested replication forks along with the helicases WRN and BLM. Heterozygous mutations mapped to the conserved nuclease or ATPase/helicase core domain of *DNA2* are linked to an adult-onset progressive myopathy characterized by mitochondrial DNA instability and oxidative phosphorylation defects^233^. At about the same time, it was reported that homozygous truncating mutations in *DNA2* were linked to Seckel syndrome, a rare disease characterized by dwarfism, severe microcephaly, and intellectual disability^234^.

DNA2 was first implicated in G4 metabolism by investigations from the Campbell and Shen labs. Biochemical studies demonstrated that purified recombinant yeast and human DNA2 proteins resolve G4 DNA substrates^235^. In a subsequent study, DNA2 mutant mice displayed elevated fragile telomeres and sister telomere associations, enhanced by the G4 stabilizing drugs TMS or TMPyP4^236^. DNA2 deficiency also caused abnormal chromosome segregation, resulting in aneuploidy-associated cancers with dysfunctional telomeres^236^. Consistent with the biological data suggesting involvement of DNA2 in G4 metabolism, purified recombinant human DNA2 was found to cleave a G4 DNA substrate in a manner dependent on its conserved nuclease (but not helicase) domain^236^. In a very recent study, DNA2 loss or chemical inhibition elevated cellular G4 levels and interfered with telomeric DNA synthesis, as demonstrated by SMARD^237^. In this work, the DNA repair complex MutSα (MSH2/MSH6) was reported to interact with DNA2 and stimulate its G4 cleavage activity at telomeres. Consistent with these biochemical data, MEFs deficient in MSH2 or DNA2 exhibit shorter and fragile telomeres, as well as increased replication stalling. Furthermore, G4-stabilizing drugs that interfere with G4 resolution-but not the G4 cleavage activity of DNA2-induce telomeric instability and compromise telomere replication, especially in cells that are deficient in DNA2 or MSH2. It will be of interest to further characterize DNA2’s action at telomeres to ascertain whether it works collaboratively with FEN1 and WRN to deal with long G-rich flaps that form G4 and require synchronous processing by helicase and nuclease activities.

## Petite integration frequency 1 (PIF1)

Petite integration frequency 1 (PIF1) is a highly conserved 5′ to 3′ ATP-dependent DNA helicase with a critical role in both nuclear and mitochondrial DNA metabolism, including DNA replication and G4 resolution^238^. In budding and fission yeast, the Pif1 DNA helicases play instrumental roles in telomere metabolism^239^. Although PIF1 mutations are not yet linked to a hereditary disease, genetic variants of PIF1 are associated with cancer^240,241^. Consistent with its important role in chromosome maintenance, PIF1 suppresses genomic instability and rearrangements associated with persistent G4s, as shown for the G4-forming human CEB1 minisatellite when inserted into yeast^242^. In yeast, Pif1 has been shown to specifically facilitate replisome progression past G4 obstacles in the lagging strand template in a manner that is dependent on its interaction with the processivity clamp PCNA^243^. Loss of PIF1 in transformed human cells results in increased fork stalling and slower DNA replication in the presence of G4 ligands^244^.

**Fig. 5:**
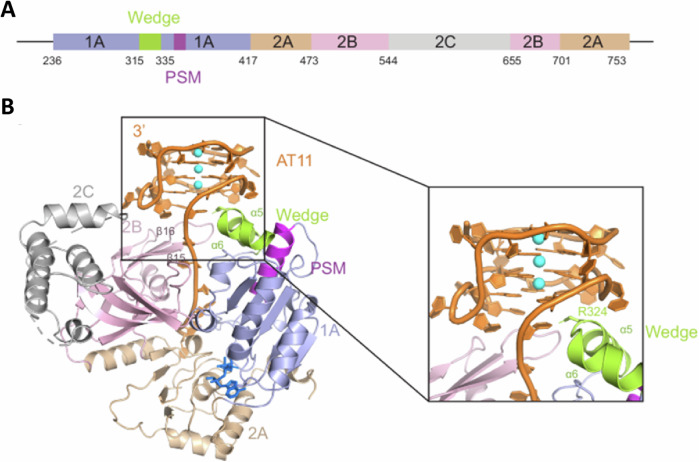
Co-crystal structure of Pif1 with G4 DNA reveals a wedge region that binds G4 DNA via a prominent interaction with the 5’ most G-tetrad. **A** Schematic of the truncated ScPif1 used in this study. **B** Cartoon representation of the crystal structure of ScPif1-G4 with the conserved domains and motifs color-coded as in **A** (1A in light blue, 2A in wheat, 2B in light pink, 2C in gray, wedge in limon, and Pif1 signature motif (PSM) in magenta). The AT11 G4 DNA is colored in orange and the ADP·AlF_4_^−^ and K^+^ ions are shown as blue sticks and cyan spheres, respectively. The key residue R324 involved in the interaction is shown as sticks. From Hong et al., *Nature Communications* (2024) 15: 6104. Ref. ^245^. The work available here: https://www.nature.com/articles/s41467-024-50575-8#rightslink is licensed under the Creative Commons Attribution 4.0 International License.

A recent co-crystal structure of *Saccharomyces cerevisiae* (sc) Pif1 with G4 DNA revealed a wedge region that binds the G-quadruplex via a noteworthy interaction with the 5′-most G-tetrad that aids in its resolution^245^ (Fig. 5). Human PIF1 shows similarities to scPif1, including sequence homology in the wedge domain^245,246^. Mutation of a wedge domain arginine that is conserved in scPif1 and human PIF1 interfered with the ability of scPif1 to resolve G4 or unwind dsDNA^245^, suggesting that scPif1 employs the same structural domain to unwind these DNA structures. Moreover, mutational inactivation of the Pif1 wedge domain interferes with Okazaki fragment processing in yeast^245^. Interestingly, a structural comparison of *Thermus oshimai* (To) Pif1 (structurally similar to scPif1) with the bovine 3′ to 5′ G4 resolving helicase DHX36 of the DEAH/RHA family led to new insights^245^. The comparison implicated ToPif1 binding to the 5′ most G-tetrad through its wedge domain versus the DHX36-specific motif (DSM), previously demonstrated to play a role in DHX36 binding of parallel-stranded G4^247^. This led Hong et al. to suggest differences in protein structure-based mechanisms for G4 recognition by the helicases Pif1 and DHX36^245^, which may contribute to their opposite translocase polarities and G4 substrate specificities.

Given the G-rich character and G4 formation potential of the human telomeric hexanucleotide TTAGGG repeat sequence, it was of interest to assess whether PIF1 plays a direct role in telomere maintenance. PIF1 was found to associate with the enzyme telomerase that extends DNA at chromosome ends in human cells^248^ and has been shown to inhibit telomerase activity^249^; however, a direct relationship of PIF1 to telomeric G4 was not assessed in either study. Nonetheless, a seminal study by Paeschke et al. showed that human PIF1 expressed in *S. cerevisiae* suppressed telomere lengthening and G4-associated DNA damage^250^, suggesting a role of the conserved Pif1 helicase in telomeric G4 metabolism. Further studies addressing how PIF1’s action at telomeric G-quadruplexes is coordinated with other proteins that bind chromosome ends in telomerase-positive cells or telomerase-negative cells, the latter of which preserve their chromosome ends via the ALT pathway, are warranted.

## DEAH-box helicase 36 (DHX36)

**Fig. 6:**
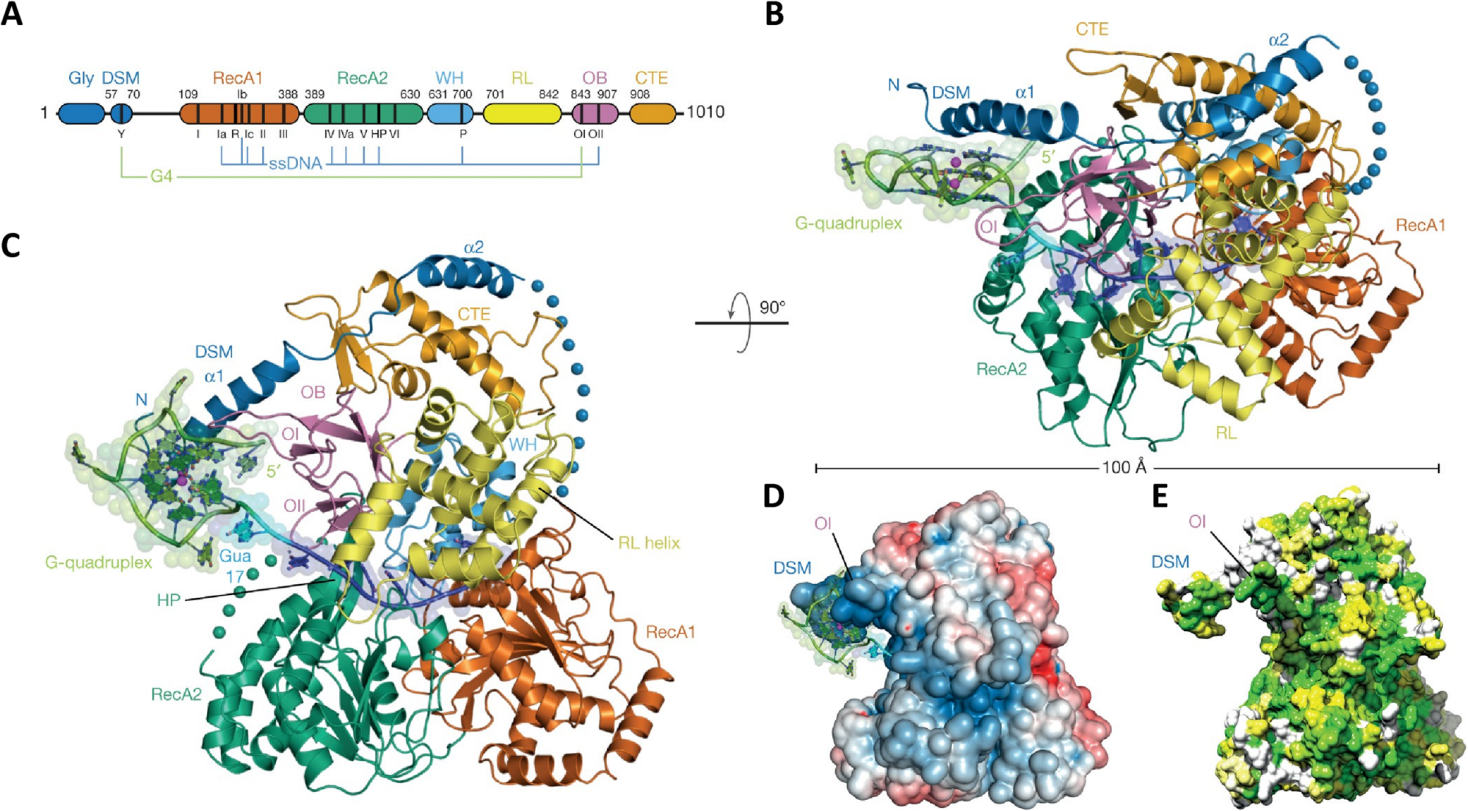
Co-crystal structure of DHX36 with parallel G4 DNA demonstrates a specific interaction with the G4 structure. **A** Domain organization; G-quadruplex (G4)- and ssDNA-interacting regions indicated. **B** Cartoon representation of the co-crystal structure of DHX36 bound to DNA^*Myc*^, colour-coded as in **A**. Spheres denote two disordered segments (blue, 20 and 53 residues in the crystallization construct and wild-type, respectively; green, 13 residues). OB loops I and II (OI and OII) contact DNA. **C** As in **B**, rotated by 90°. **D**, Electrostatic potential calculated with DNA omitted from the co-crystal structure (blue to red, ±5 *k*_B_*T*). **E**, Phylogenetic conservation among 250 DHX36 orthologues (white to green, least to most conserved). Figure obtained by permission from the publisher of *Nature* via RightsLink process; Chen et al., *Nature* (2018) 558, 465-469. Ref. ^247^ The Licensor’s permission is acknowledged; RightsLink Printable License.

The aforementioned DHX36 (RHAU/G4R1) is a DNA/RNA helicase with key roles in embryonic development, spermatogenesis, and hematopoiesis^251–254^. DHX36 was shown to serve as a prominent G4 resolvase in extracts derived from HeLa cells^255^ and can resolve DNA G4s through a combination of DNA translocation and unfolding mechanisms^247,256^. A co-crystal structure of bovine DHX36 with DNA derived from the Myc promoter parallel G4-forming sequence flanked by a short ssDNA tail demonstrated the importance of a DSM α-helix that with an OB-fold-like subdomain forms a specific interaction with the G4^247^ (Fig. 6). The structural data along with SM FRET studies of G4 unfolding by recombinant wild-type DHX36 and DHX36 variants harboring site-directed mutations in the DSM led Chen et al. to propose a model in which DHX36 pulling on the 3′-single-stranded tail adjacent to the G4 causes rearrangements leading to G4 disruption by 1-nucleotide steps.

Several years ago, Knipscheer and colleagues showed using a reconstituted Xenopus egg extract system to study DNA replication that DHX36 cooperates with FANCJ to resolve G4s in a multi-step mechanism^19^. First, DHX36 enables the replicative helicase CMG to bypass G4 obstacles on the leading strand, therefore avoiding fork stalling. Second, the G4 structure is resolved by FANCJ. G4s on the lagging strand do not stall CMG; however, DHX36 and FANCJ are still required for their resolution. The precise molecular mechanism for CMG bypass currently remains unclear and is an important topic for future studies. Consistent with an important role of DHX36 in genome metabolism, loss of DHX36 results in genomic instability, hypersensitivity to G4-stabilizing compounds, and dysregulation of innate immune gene expression^257,258^. In a very recent study, the Knipscheer lab and collaborators utilized the Xenopus egg extract system and mammalian cell models to study genomic G4 landscapes in which a G4 DNA lesion activates the signaling kinases ATM and ATR and promotes subsequent RNA strand invasion mediated by BRCA2 and RAD51, such that an RNA-DNA hybrid forms opposite to the G4^197^. The coincidence of G4 and R-loop structures induces inappropriate transcripts, replication stress, and destabilization of the genome. In response to this, the helicases DHX36 and FANCJ resolve the G4, which enables structure-specific incision specifically by the XPF-ERCC1 nuclease that aids in G-loop disassembly, ultimately shaping the G4-R-loop landscape in eukaryotic systems. Given that endogenous G4s are abundant, and cancer and developmental abnormalities are associated with molecular defects in genes controlling G4-R-loop frequency (e.g., XPF-ERCC1 mutations linked to multiple hereditary disease states, including Xeroderma pigmentosum combined with Cockayne syndrome or FA^259^), it is of great priority to establish if G-loop formation drives distinct signatures of genomic instability characteristic of the respective rare diseases.

## Importance of G4 in neuronal cells, neurodegeneration, and neurodevelopment

Although this review focuses primarily on the effects of G4 on cellular DNA replication, experimental evidence supports the hypothesis that G4s can affect gene expression. For example, G-rich promoter elements can form G4, especially under conditions of G4 ligand stabilization, causing a change in downstream gene expression. Indeed, rare diseases such as WS or BS that are defective in G4-resolving helicases display changes in their transcriptional profiles, particularly in genes with G-rich promoters or first introns^260–262^.

Ligand-induced G4 stabilization has been shown to impact phenotypes related to brain pathology in neurons or neuronal stem cells that may reflect changes in gene expression or induced DNA damage. An early demonstration of this was evidenced by work from the Tsvetkov lab in which the authors found that PDS downregulates transcription of *Brca1* and promotes DNA DSBs in cultured neurons, as well as decreases neuronal survival and damages synapses^263^. In subsequent work, Moruno-Manchon et al. determined that pharmacological stabilization of G4 in neurons caused a significant reduction in expression of the *Atg7* gene, which plays a paramount role in autophagy initiation, that has been found to decrease with aging^264^. The group demonstrated that brain samples of aged mice display an enrichment of immunostained G4 in vivo; furthermore, mice injected with the G4 ligand PDS developed recognition memory defects and an accumulation of lipofuscin, a marker of oxidized proteins and lipids in aged brains^265^.

More recently, Escarcega et al. assessed via genome-wide gene expression analysis (RNA-Seq) the effect of PDS exposure in primary cultured neurons^266^. Transcriptional changes were observed for pathways involving p53 signaling, immune response, learning, memory, and cellular senescence. PDS-induced up-regulation of the *Pirh2* gene encoding an E3 ubiquitin ligase implicated in the DNA damage response caused DNA DSBs in neurons, accompanied by down-regulation of the DDX21 RNA helicase known to resolve RNA G4^267,268^. These studies evidence the significant influence of G4 structures in alteration of neuronal gene expression and DNA damage formation, impacted by G4-resolving helicases.

While there is compelling evidence for the utility of G4 ligands to regulate gene expression, recent work suggests that there may be some caveats to interpretations from cell-based studies. The Tsetkov lab recently reported opposite effects of G4 ligands on gene expression in human astrocytes, implicated in brain disorders, that is dependent on the drug’s ability to *stabilize* or *destabilize* cellular G4s^269^. Thus, new opportunities to switch on or off G4 regulatory systems are on the horizon, opening new avenues for G4-directed therapeutic strategies. While it is tempting to solely implicate transcriptional aberrations and damaged genomic DNA in neuron pathology associated with brain disease, aged neurons themselves can attempt DNA replication^270^. Replicative DNA synthesis was observed to precede neuronal cell death in Alzheimer’s Disease^271^. Conversely, very recent work provided evidence that DNA replication fork speed during embryonic cortical neurogenesis can have an impact on adult mouse behavior^272^.

The rare hereditary disorder X-linked dystonia parkinsonism (XDP) provides another illustrative case of an age-related neurodegenerative disease with a G4 connection. XDP is characterized by progressive motor dysfunction and extensive neurodegeneration in the basal ganglia^273,274^. The Richter lab and collaborators collected experimental evidence from in vitro studies that G4s within a SINE-VNTR-Alu (SVA)-type retrotransposon that inserts into a human gene encoding TATA-binding protein-associated factor 1 (TAF1), a partner of RNA polymerase II, inhibits polymerase amplification; furthermore, they showed by BG4 ChIP that G4s are present in XDP patient-derived fibroblasts^275^. G4 ligand exposure causes aberrant *TAF1* gene expression in the XDP cells, suggesting that G4 is the culprit that might be targeted using G4-unfolding therapeutics. Disease onset of XDP correlates inversely with age and positively with polymorphic expansion of hexameric repeat elements within the SVA retrotransposon harbored in the *TAF1* gene^276^, suggesting that the contiguous G4s potentiate aberrant effects on transcription and genomic instability, possibly via a replication-linked process, but the precise mechanism remains to be determined.

Because XDP is attributed to mutations in the *TAF1* gene residing on the q-arm of the X chromosome, males with bi-allelic mutations are fully affected, whereas females, as heterozygous carriers, are less severely impacted^277^. As suggested by Escarcega et al., G4-driven pathology in XDP may in part be sex-based^278^. Emerging evidence from other investigations also suggests that sex differences in replication or DNA repair defects exist^279,280^. Further study is warranted to ascertain if G-quadruplexes have consequences that are dependent on sex, and if helicases play a role. For example, the RNA transcript binding G4 helicase DDX3X^281^ is implicated in the rare neurodevelopmental disorder known as DDX3X syndrome in which affected individuals, mostly females, display intellectual disability and complex comorbidities^282^. The *Ddx3x* gene, located on the X chromosome, and its Y chromosome homolog *Ddx3y* display a complex relationship with one another that potentially influences sex dissimilarities in senescence and aging^278^. Whether G4 plays a role in the apparent sex-dependent gerontological differences remains to be seen.

## Discussion

**Fig. 7:**
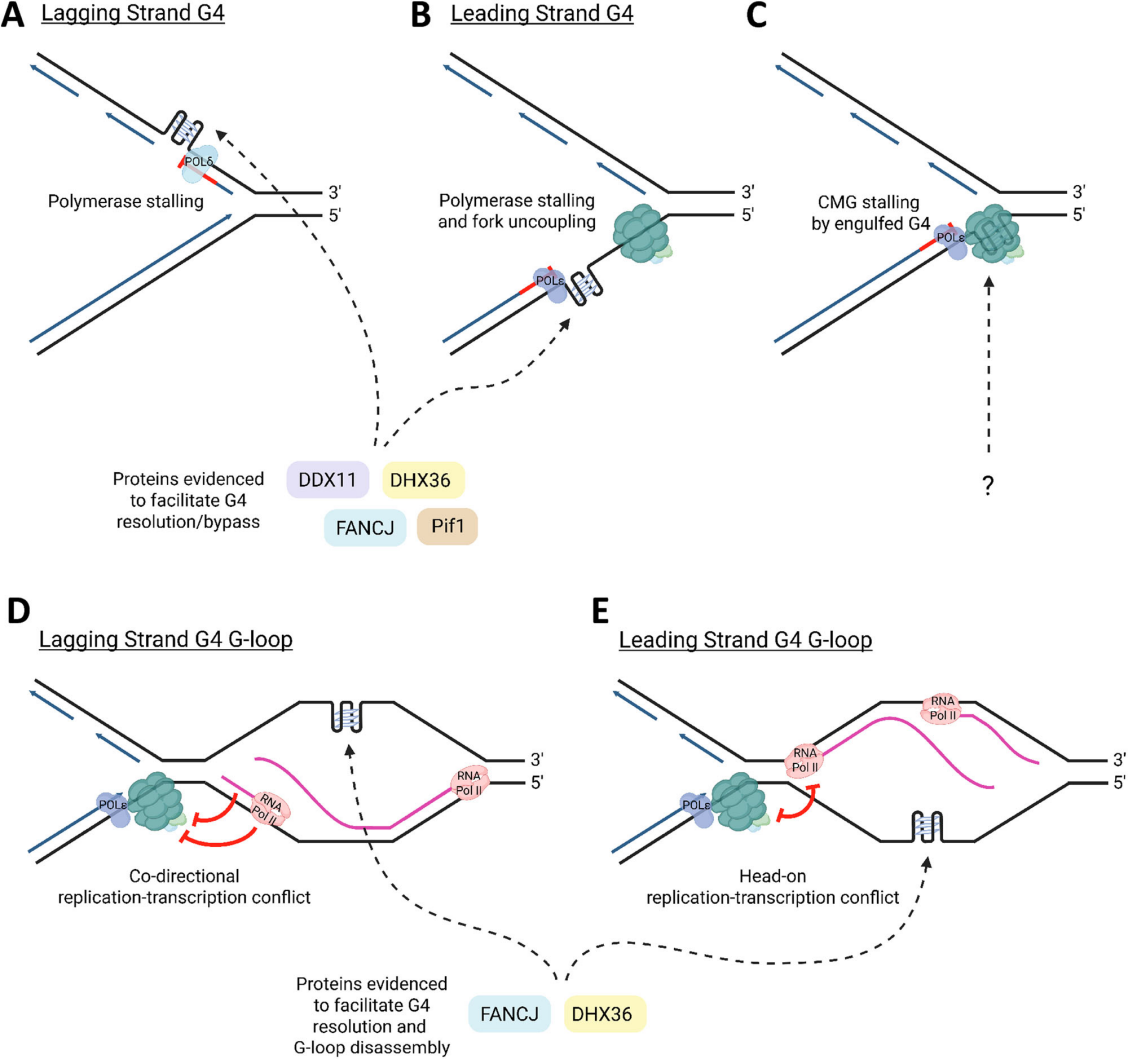
Auxiliary G4-resolving helicases that facilitate DNA synthesis past replisome-stalling G4 structures. Some of the proposed targets of specialized DNA helicases on G4 structures emerging at the replication fork are shown. Proteins experimentally evidenced to promote replisome progression past lagging (**A**) and leading (**B**) strand polymerase-stalling G4 structures formed by ssDNA exposure during replication through G4 resolution or bypass are featured. Note that G4 that forms from transient ssDNA between Okazaki fragments (**A**) or created in the wake of CMG parental duplex unwinding (**B**) are depicted. The mechanism by which replication resumes following stalling caused by CMG helicase engulfment of a pre-formed G4 (**C**) is a notable question for future study. Lagging strand G4 G-loop (**D**) and leading strand G4 G-loop (**E**) arising from transient ssDNA created by R-loop formation are stabilized by G-quadruplexes and result in co-directional and head-on replication-transcription conflicts, respectively. Created in BioRender. Herr, L. (2025) https://BioRender.com/lvrqjly.

In this review, we have highlighted experimental evidence detailing pathways and mechanisms whereby genetic mutations linked to rare diseases cause elevated G4-induced replication stress. Fundamentally, G4 structures that form in guanine-rich sequences found in the leading or lagging strand template may interfere with efficient DNA synthesis, posing detrimental consequences for genomic stability (Fig. 7). Among the potential mechanisms of G4-induced replication stress, interference of DNA replication can occur by impeding smooth polynucleotide synthesis by DNA polymerases or trapping the replicative CMG helicase complex. However, G4 structures that form during nascent synthesis of the newly manufactured guanine-rich strand may also impair genomic stability by interfering with processing of key DNA replication intermediates such as 5′ flaps (Fig. 4). In all of these scenarios, the transient formation of ssDNA in guanine-rich stretches that can assemble into stable G4 structures is predicted to be the primary driver of G4-induced replication stress. Genetic defects resulting in the dysfunction of DNA replication or repair proteins that either suppress the formation of G4s or unfold/resolve G4s that do form can seriously compromise replication processivity or fidelity.

It is reasonable to predict that mechanisms which either promote or diminish the stability of G4 would alter cellular genome homeostasis. As discussed in this review, drugs that promote or stabilize G4 are often used as tools to probe the consequences of G4 abundance in cells. Such G4-interactive drugs are proposed candidates for anti-cancer or anti-viral therapies. Conversely, proteins that discourage the formation or persistence of G4s contribute to suppression of their deleterious consequences, especially during DNA replication. A prominent class of enzymes that fit this description is the specialized G4-resolving helicases, such as those that belong to the RECQ or Fe-S cluster helicase families. A formidable question is how specialized G4-resolving helicases gain access to a G4 structure engulfed by the CMG replicative helicase complex. Presumably, protein-protein interactions of factors stably associated with the replication fork play a significant role in the recruitment of G4 resolvases that act as auxiliary factors in situations of G4-induced replication stress.

DNA translocases that remodel stressed replication forks may play a role in the response to G-quadruplexes encountered during replicative DNA synthesis; however, it remains to be definitively shown that fork regression is elicited upon G4-induced stalling. An area that requires more study is the mechanisms whereby genome-specific loci prone to G4 formation are dealt with in a regional manner and if this occurs by specialized G4-metabolizing proteins, e.g., helicases acting as G4 resolvases. For example, we discussed experimental evidence for specialized mechanisms and proteins that play a role in telomeric G4 processing. Presumably, there are operational pathways for other G-rich loci, such as those found in ribosomal DNA^283^. Further studies are warranted.

It is plausible that DNA-binding proteins which do not require ATP hydrolysis for translocation may also be relevant players in G4 metabolism, including during processes occurring at the replication fork. For example, the ssDNA binding protein RPA may bind transient guanine-rich single-strands that arise during replicative DNA synthesis, preventing them from forming G4s. In this scenario, it would be of interest to assess if human cells overexpressing RPA are resistant to G4-induced replication stress imposed by G4 ligands, paralleling studies for other forms of pharmacologically induced replication stress^284–286^.

From a structure-function perspective, the helicase-G4 structures highlighted in this review have begun to suggest conserved mechanisms whereby helicase proteins already implicated in different aspects of cellular DNA replication and maintenance of genomic stability interact with and act upon G4s. The theme that a protein sub-domain specifically recognizes and binds G4s is prominent. Another take-home message is that certain G4 resolvases may operate to disassemble G4 structures by removing one nucleotide at a time. Therefore, the overly simplistic concept that a G4 resolvase pulls one leg of the four-stranded table, causing the entire G-quadruplex to collapse, may be largely inaccurate. Moreover, we know very little at this stage about whether the mechanisms of action for G4 resolution by a given helicase are unique depending on the architecture of the G4, i.e., uni-molecular or multi-stranded, parallel or anti-parallel. Given that certain helicases strongly prefer to bind and resolve a G4 of a specific architecture, it seems probable that G4-resolving helicases target defined G4 topologies that may be genome-region specific (e.g., telomeric overhangs) or context-dependent (e.g., replicative G4 bypass or gene promoter activation).

**Fig. 8:**
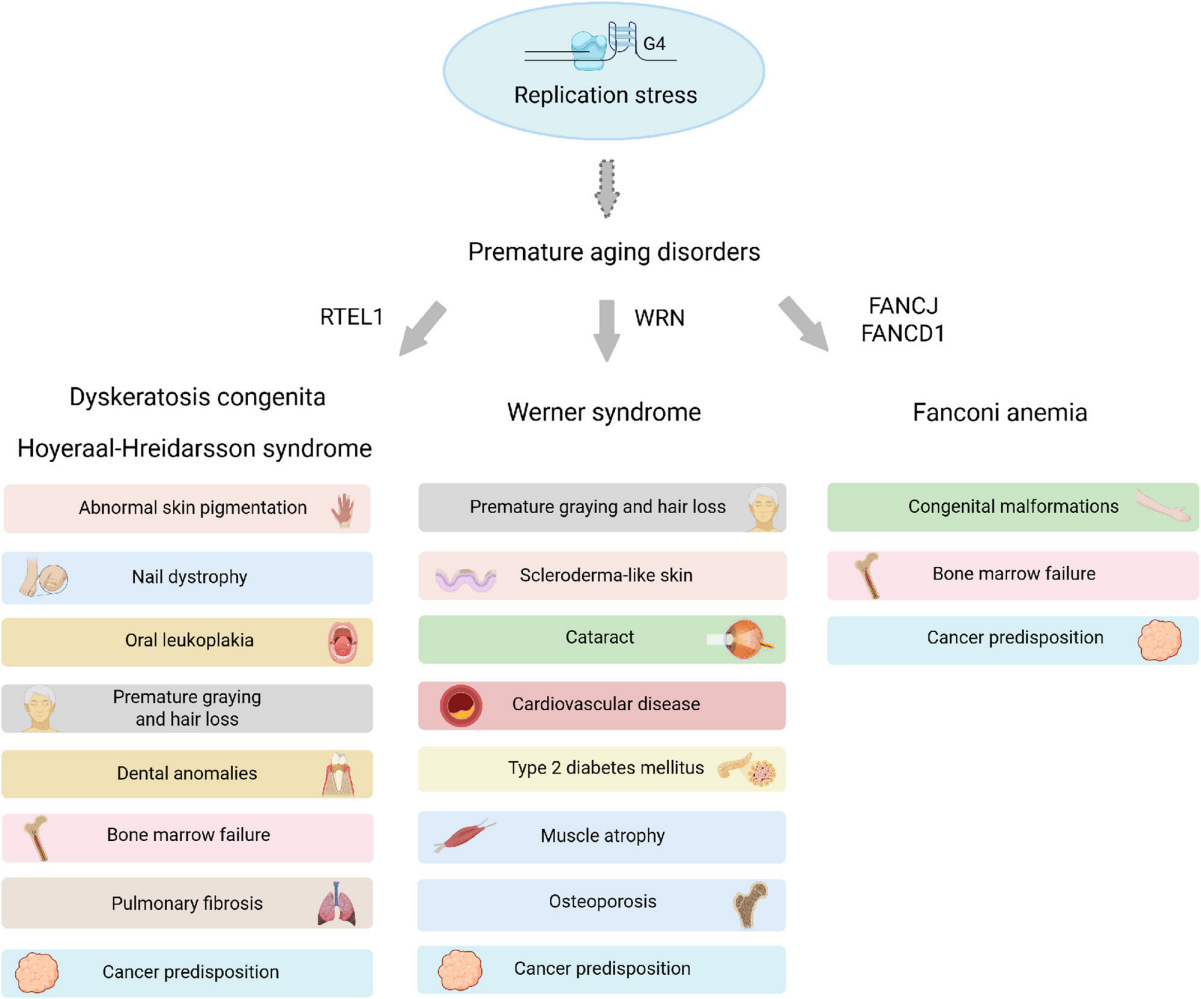
Representative diseases of accelerated aging with molecular defects in the replication stress response and G4 DNA metabolism. Certain hereditary disorders are linked to mutations in genes encoding proteins involved in G4-related DNA transactions in cells or model organisms. However, the proteins defective in these genetic diseases play broader roles in genome biology such as the replication stress response or DNA repair. Created in BioRender. Rossi, M. (2026) https://BioRender.com/3vstaqa.

In terms of their relevance to rare diseases, cellular responses and mechanisms that suppress G4-induced replication stress emerge as primary pathways to suppress genomic instability, as discussed throughout this review. Although G4 structures clearly influence replication dynamics and genomic stability, the extent to which G4 processing per se drives human rare diseases remains to be fully elucidated and requires further investigation. Several representative rare diseases associated with molecular defects in G4 DNA metabolism and elevated replication stress that present with accelerated aging or senescence phenotypes are shown in Fig. 8. These highlight the potential physiological consequences of impaired G4 physiology, but direct causal links remain to be firmly established. For example, ascertainment of a causal relationship of G4s to inflammation in human diseases, neurodegeneration, and accelerated aging is of interest^287,288^.

A perhaps still under-appreciated issue is the mechanistic importance of the ligand-induced stabilization of G-quadruplexes in the activation of the ataxia telangiectasia and Rad3-related (ATR)-dependent ataxia telangiectasia mutated (ATM) signaling pathway^289^. ATR kinase, known to respond to replication stress, was found to become activated by cellular exposure to the G4-binding compound RHPS4, a treatment that interfered with telomeric DNA synthesis and the binding of shelterin proteins to chromosome ends. *ATR* mutations are linked to the rare genetic disorder Seckel syndrome (SS), characterized by neurological defects, microcephaly, and dwarfism (Seckel Syndrome - Symptoms, Causes, Treatment | NORD), whereas *ATM* mutations are linked to the rare disorder ataxia-telangiectasia (A-T) that affects the nervous and immune systems, and other body systems as well (Ataxia Telangiectasia - Symptoms, Causes, Treatment | NORD). While the clinical importance of G4s in SS or A-T remains to be fully characterized, it seems probable that G4-induced replication stress will come into play given the importance of the ATR/ATM kinases in the DNA damage response associated with perturbed cellular DNA replication. Recently, a successful experimental approach utilizing induced pluripotent stem cells to correct the splicing defect attributed to a hypomorphic *ATR* mutation responsible for SS has suggested a potential avenue for a therapeutic recovery of SS neuronal defects^290^, prompting interest in whether a restored response to G4-induced replication stress is one of the factors underlying the positive outcome.

A better understanding of the roles of G4s in cellular senescence, inflammation, immunity, and neuronal pathway function will surely aid in the characterization of molecular pathology underlying rare diseases. Determination of the redundant, partially redundant, or unique functions of G4-metabolizing proteins may help to improve diagnosis of rare diseases according to their molecular-genetic defects and the underlying causes of G4-dependent phenotypes. While there is much interest in G4 ligands as drugs to combat cancer and viral infections, the knowledge gained from further characterization of G4-specific pathways can also be leveraged to enhance therapeutic strategies to treat rare diseases. Next-generation strategies based on nucleic acid-mediated correction of gene expression^291^, including those that utilize CRISPR-directed G4-interactive proteins and ligands^292^, represent new opportunities for rare disease treatment strategies. Elucidating the roles of G4 metabolism in molecular and cellular biology will allow for a better understanding of related rare diseases and foster the development of therapeutics to treat cancer, viral infections, and genetic disorders.
